# Bin2 Is a Membrane Sculpting N-BAR Protein That Influences Leucocyte Podosomes, Motility and Phagocytosis

**DOI:** 10.1371/journal.pone.0052401

**Published:** 2012-12-20

**Authors:** María José Sánchez-Barrena, Yvonne Vallis, Menna R. Clatworthy, Gary J. Doherty, Dmitry B. Veprintsev, Philip R. Evans, Harvey T. McMahon

**Affiliations:** 1 MRC Laboratory of Molecular Biology, Cambridge, United Kingdom; 2 Cambridge Institute for Medical Research, University of Cambridge School of Clinical Medicine, Addenbrooke’s Hospital, Cambridge, United Kingdom; Université de Genève, Switzerland

## Abstract

Cell motility, adhesion and phagocytosis are controlled by actin and membrane remodelling processes. Bridging integrator-2 (Bin2) also called Breast cancer-associated protein 1 (BRAP1) is a predicted N-BAR domain containing protein with unknown function that is highly expressed in leucocytic cells. In the present study we solved the structure of Bin2 BAR domain and studied its membrane binding and bending properties *in vitro* and *in vivo*. Live-cell imaging experiments showed that Bin2 is associated with actin rich structures on the plasma membrane, where it was targeted through its N-BAR domain. Pull-down experiments and immunoprecipitations showed that Bin2 C-terminus bound SH3 domain containing proteins such as Endophilin A2 and α-PIX. siRNA of endogenous protein led to decreased cell migration, increased phagocytosis and reduced podosome density and dynamics. In contrast, overexpression of Bin2 led to decreased phagocytosis and increased podosome density and dynamics. We conclude that Bin2 is a membrane-sculpting protein that influences podosome formation, motility and phagocytosis in leucocytes. Further understanding of this protein may be key to understand the behaviour of leucocytes under physiological and pathological conditions.

## Introduction

An ability to remodel the plasma membranes is critical for cell migration and shape plasticity. At the plasma membrane dynamic changes in the membrane composition are required to position adhesion molecules and guidance molecules. Also the extension of protrusions in the form of filopodia/lamellipodia or podosomes requires coordinated changes in cytoskeletal architecture and membrane shape. Bin2 (also called BRAP1) is a putative membrane remodelling protein whose function is unknown but high levels of mRNA are found in leucocytes and leukaemic cells [1].

At its N-terminus, Bin2 has a predicted N-BAR domain while the C-terminus (comprising 328 of the 565 amino acids in human Bin2) is rich in glutamates, serines and prolines (41, 58 and 43, respectively) with no strong homology to other proteins. Both the N-terminus and especially the C-terminus are heavily phosphorylated according to the data in www.phosphosite.org. BRAP2, whose name may suggest similarity, is unrelated in sequence. Across species the C-terminus of Bin2 is most divergent, with 43% and 15% sequence identity between human and rat or dog sequences, respectively. It is also found in frog (Xenopus) and zebrafish (Danio) but is not found in worms or flies.

The N-BAR domain of Bin2 is positioned within the BAR domain superfamily of membrane remodelling domains alongside the N-BAR domains of amphiphysins and endophilins [2]. The prototypical BAR module is a rigid, elongated, banana-shaped dimer as seen for Amphiphysin [2] and Endophilin [3], [4]. Inverse BAR modules and F-BAR modules are variations on the basic BAR architecture [5], [6], [7]. In all cases the structural architecture of the membrane binding face is imprinted on the membrane. BAR domains are frequently associated with other lipid binding domains in proteins. One such association occurs in a subgroup of BAR family members that have an additional N-terminal amphipathic helix which inserts in a shallow manner into the bilayer and induces positive curvature, that is consistent with, and stabilized by the BAR domain architecture [4], [8]. These N-BAR domains exert both intrinsic (amphipathic helix) and extrinsic forces (BAR module) on the membrane to promote changes in membrane curvature.

In this study, we investigated the structure and function of Bin2 in leucocytes. We solved the structure of its BAR domain by X-ray crystallography and show both *in vitro* and *in vivo* that it can tubulate membranes. We show that two mutations in its dimeric interface limit its ability to bind membranes. We confirm that Bin2 is enriched in leucocytes where it localizes to podosomes, the leading edge of migrating cells and to the macrophage phagocytic cup. Bin2 localizes to the ring-like structure around the actin core of podosomes in an N-BAR-dependent manner whilst its C-terminal unstructured tail permits Bin2 interaction with α-PIX and Endophilin A2. Bin2 knockdown results in impaired monocyte migration but enhanced macrophage phagocytic function. Taken together, these data suggest that Bin2 is a *bona fide* member of the N-BAR protein family that has an important role in leucocyte morphogenesis.

## Results

### Bin2 is Specifically Expressed in White Blood Cells

**Figure 1:**
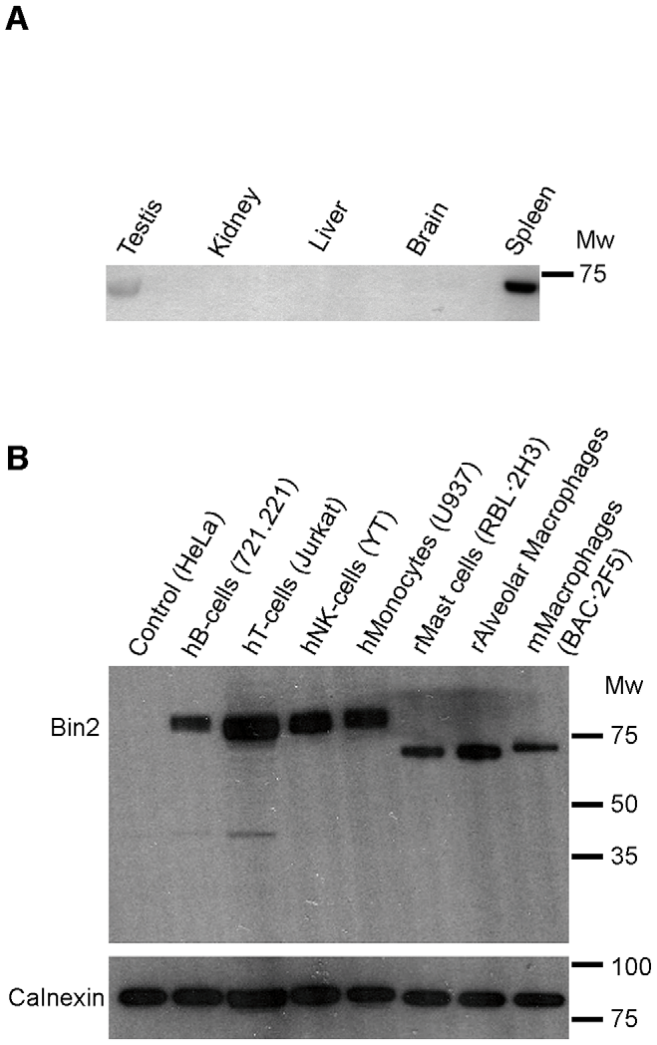
Bin2 is mainly expressed in hematopoietic cells and leucocyte-enriched tissues. *A* : Expression of Bin2 in rat tissues. Cell homogenates from the indicated tissues were resolved by SDS-PAGE and immunoblotted with our polyclonal anti-Bin2 antibody (BACT). ***B***: Endogenous expression of Bin2 in different leucocytic cell lines. An anti-calnexin antibody was used as a loading control. Molecular weight markers (Broad Range, Promega) are indicated.

A rabbit polyclonal antibody was raised against human Bin2 C-terminus. Bin2 protein was detected in the spleen but was absent from non-leucocyte-rich tissues such as the liver and kidney (Figure 1A and Figure S1). This is consistent with previous northern blot analyses suggesting that Bin2 is predominantly expressed in hematopoietic cells [1]. Further analysis showed that Bin2 is expressed in both myeloid and lymphoid lineages, suggesting that this protein may play a role in all leucocyte lineages (Figure 1B). Leucocytes are highly motile and undergo many complex membrane-deformation processes during cell migration and the internalisation of antigens. They are rich in podosomes, which are actin-rich adhesive structures thought to play an important role in leucocyte chemotactic migration.

### The Structure of Bin2 BAR Domain

**Figure 2:**
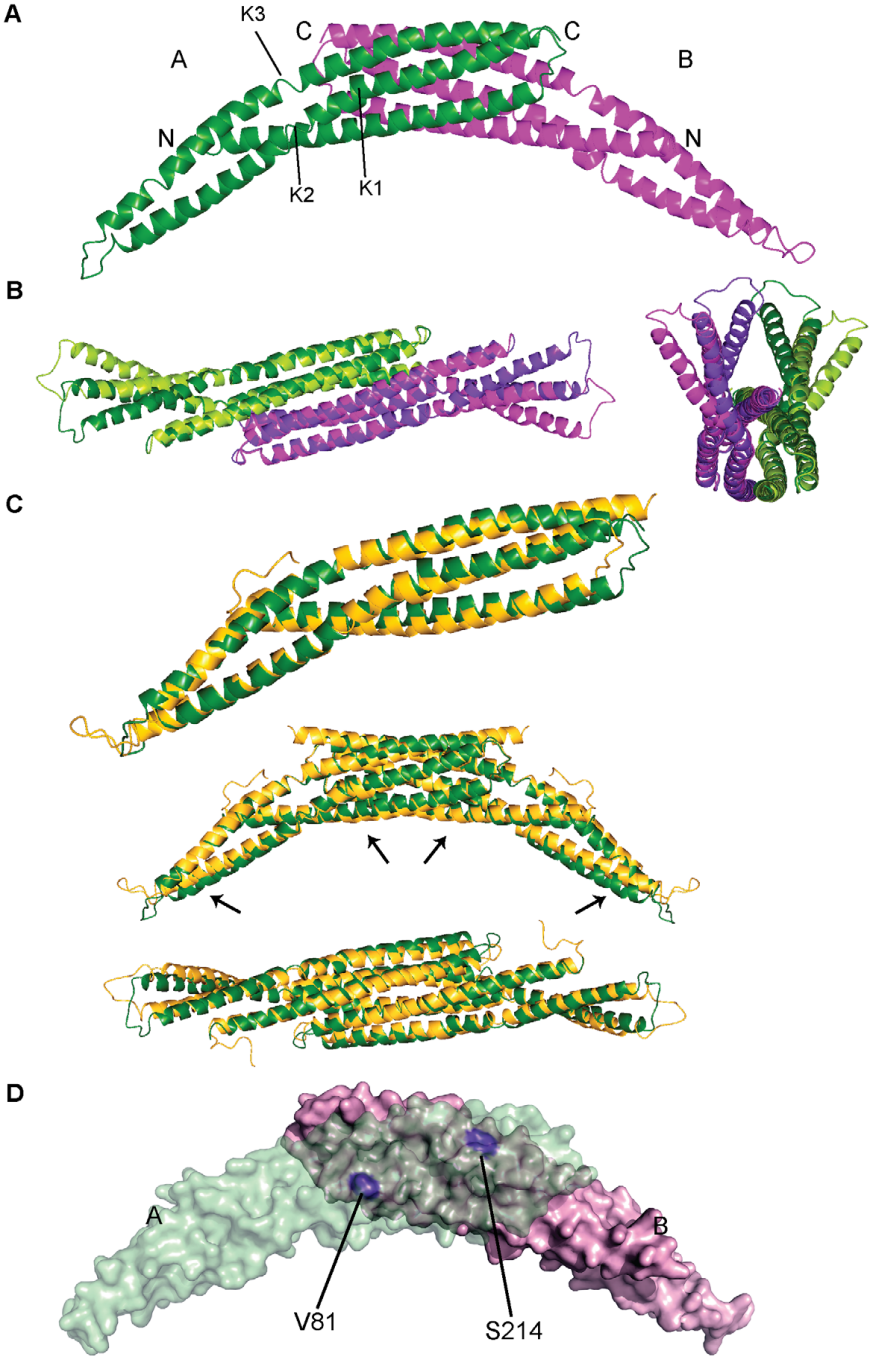
The structure of the hBin2 BAR domain. *A* : Ribbon representation of the dimeric crystal structure found in the asymmetric unit. Subunit A is depicted in green and B in purple. Kinks in helix 2 (K1 and K2) and helix 3 (K3) are indicated. ***B***: The different conformation of the BAR ends. Ribbon representation of the dimer superimposed on itself by a 180° rotation, so that subunit A is superimposed on B and vice versa. The central region, Cα atoms from amino acids 38–131 and 197–238 were used for the superimposition. The dimer and its rotated version are shown in dark and light colours respectively, green and purple as in (A). A view of the concave face is show on the left and the “end-on” view on the right. ***C***: Comparison of hBin2 and dAmph BAR structures. On the top: ribbon representation of the superposed monomers. hBin2 subunit A in green and dAmph in yellow. In the middle and bottom: superimposition of the BAR dimers showing the slightly smaller curvature of Bin2 defined by the concave surface. Arrows indicate the regions that contribute to the differences in curvature. A side view is shown in the middle and a view of the concave surface on the bottom. ***D***: Molecular surface representation of hBin2 BAR dimeric crystal structure showing the dimerization interface. Subunit A is depicted in transparent green and subunit B is depicted in pink. Residues V81 and S214 located on the surface of protomer B are highlighted in violet.

To approach an understanding of Bin2 function we initially characterised the N-terminal region, which has high sequence homology with that of other BAR domain proteins. The structure of the predicted Bin2 BAR was determined by MIRAS using a native crystal and a Se-Met derivative (Table 1). A dimer was present in the asymmetric unit and the general architecture of the dimer resembled an elongated banana as found for other BAR modules (Figure 2A). Each monomer was a coiled-coil, formed by three long, bent helices. Helix 1 comprised amino acids 38 to 88, helix 2 (from 95 to 159) was kinked in two places, at residues Asp119 and Gln131 (labelled as K1 and K2 in Figure 2A), and helix 3 (residues 169 to 238) was disrupted at Pro196 (K3 in Figure 2A). The kink in helix 3 was the only one that is conserved among its structural homologues, human Amphiphysin2 (hAmph2), fly Amphiphysin (dAmph), and rat Endophilin A1 (rEndoA1), and was always mediated by a proline, which usually also mediates the kink in helix 2 except in Bin2 [2], [4], [9].

**Table 1:** Data collection, phasing and refinement statistics.

Crystal	Se-Met 1Peak(P)		Se-Met 1Inflexion (Ip)		Se-Met 1Remote (Rm)	Se-Met 2Peak(P)		Native
Wavelength Å	0.9791		0.9792		0.9756	0.9791		0.9791
Space Group	P2_1_2_1_2_1_					P2_1_2_1_2_1_		P2_1_2_1_2_1_
a b c	81.0 81.6 82.3					81.4 81.7 81.9		79.1 81.3 81.1
α β γ	90 90 90					90 90 90		90 90 90
Resolution (Å)	58.0–3.15		57.8–3.15		47.1–3.15	47.1–3.3		57.5–2.53
**Rmerge (%)	11.6 (73.1)		8.4 (56.8)		8.5 (59.1)	12.3 (72.2)		8.3 (38.6)
I/σ(I)	4.1 (1.0)		6.3 (1.3)		5.7 (1.3)	3.7 (1.1)		6.4 (1.9)
Completeness(%)	99.9 (99.9)		92.8 (92.8)		92.9 (92.9)	99.8 (99.8)		94.9 (96.5)
Multiplicity	12.9 (13.5)		3.3 (3.3)		3.3 (3.4)	12.5 (13.1)		4.6 (4.6)
Ano Compl. (%)	99.9 (100)		79.8 (80.6)		80.1 (80.7)	99.8 (100)		
Ano Multipl.	6.6 (6.6)		1.9 (1.9)		1.9 (1.9)	6.7 (6.8)		
**Phasing power**								
Isomorphous/anomlous	0.49/0.92		0.60/0.78		−/0.56	0.35/0.90		0.88/−
**Phasing**	**N Acentric (A)**	**FOM Acentric**				**N Centric (C)**	**FOM centric**	
Overall	14408	0.296				2130	0.306	
**Refinement**								
Resolution (Å)								57.41–2.53
No. Reflections								14986
Test set size (%)								5.4
Rwork (%)								19.6(22.9)
Rfree (%)								25.2(23.8)
Twin Operator								k h -l
Twin fraction (%)								11.2
No. Atoms (Non-H)								3393
<B> (Å^2^)								59.8
R.m.s. deviations								
Bond length(Å)								0.014
Bond angles (°)								1.74

* Highest resolution shell (in Å) shown in parenthesis. Values for Se-Met 1 (3.15–3.32), Se-Met 2 (3.30–3.48) and Native (2.53–2.73).

** Rmerge = Σ_hkl_ |I_hkl_ - <I_hkl_>|/Σ_hkl_ I_hkl._

Different conformations for the extended regions of the dimer wings were observed for each monomer in the dimer. Concretely, the region between kinks K2 and K3 (amino acids 131 to 196) showed different conformations in each monomer and this was likely due to crystallographic contacts. The movement can be described as a lateral displacement of helices 2 and 3 (Figure 2B). This does not affect the curvature defined by the concave face of the dimer, which would be consistent with a scaffolding mechanism for membrane binding, where a defined membrane binding interface is needed [8]. The loops at the extremities of the BAR wings were poorly ordered.

The overall fold of hBin2 BAR was very similar to that of Amph BAR [2], [9] consistent with the high sequence homology with dAmph and hAmph2 (N-BAR sequence identity of 37% and 62%, respectively). The concave face can accommodate a circle of diameter 170 Å, which was slightly smaller than that of Amph due to the way the protomers intersected and the distinct helix kinking (Figure 2C).

The dimerization interface of hBin2 (Figure 2D) was smaller than those of dAmph or hAmph2. The buried surface area per monomer in hBin2 was 1930 Å^2^, whilst that for dAmph was 2400 Å^2^. While in hBin2 there were only 5 intermolecular hydrogen bonds, there were up to 15 in dAmph. Hydrophobic contacts important for dimer formation and stability are more abundant in dAmph. These differences are consistent with the longer dimerization interface in dAmph. Despite these differences, the Kds for dimerization in solution were similar, with the hBin2 dimer being 7 µM (see Methods and Figure S2), compared with the dAmph dimer of 6 µM [2].

### Bin2 N-BAR Interaction with Membranes

**Figure 3:**
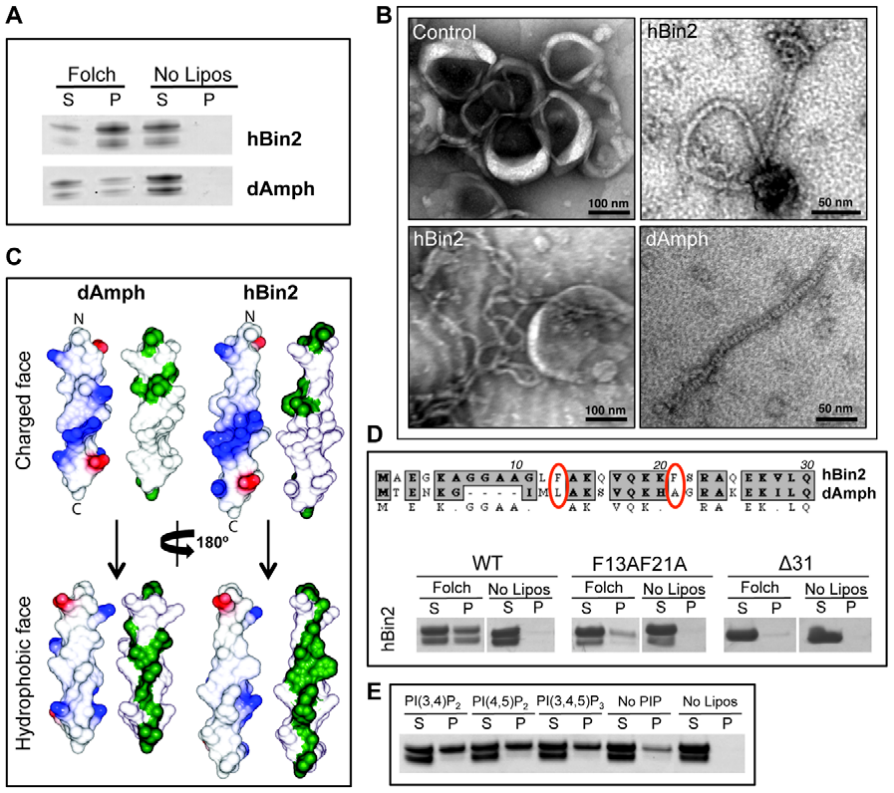
*In vitro* binding of hBin2 N-BAR to membranes. ***A***: hBin2 N-BAR binds more tightly to membranes that dAmph, despite the high sequence homology. Coomassie-stained gels of cosedimentation assays of hBin2 and dAmph N-BARs, with Folch liposomes. S, supernatant and P, pellet. ***B***: hBin2 tubulates liposomes in a similar fashion to dAmph N-BAR. Electron micrographs of Folch liposomes tubulated by hBin2 (12–15 nm diameter) or dAmph N-BARs (∼12 nm diameter). ***C***: Molecular surface representation of modelled N-terminal amphipathic helices. In blue, positive charges, in red, negative charges and in green, hydrophobic residues. ***D***: Bin2 H0 helix is essential for membrane binding and bulky hydrophobic residues of Bin2 H0 helix contribute to the enhanced affinity to membranes compared with dAmph. Sequence alignment of hBin2 and dAmph H0 helix. Bulky hydrophobic amino acids are highlighted in red. Liposome cosedimentation assay as performed in (A) with hBin2 N-BAR amphipathic helix-mutants: F13A,F21A and H0 deletion mutant (Δ31). ***E***: Bin2 has no specificity for phosphoinositides (PIPs) but prefers membranes enriched in PIPs. Lipid cosedimentation assays with synthetic liposomes (70% PC, 30% PS, 10% cholesterol, 1% PIPs).

Membrane binding and curvature sensing/inducing are the defining characteristic of BAR domain proteins. We studied the membrane binding ability of hBin2 and dAmph N-BAR domains by lipid co-sedimentation assays. Under the experimental conditions, hBin2 bound to Folch liposomes with higher affinity than dAmph (Figure 3A). Both proteins were slightly degraded on the N-terminus after purification (see Methods). Using electron microscopy of protein-lipid mixtures we showed that Bin2 tubulated liposomes more efficiently than dAmph. The diameter of the tubules produced by hBin2 varied from 12–18 nm, with the most abundant being ∼15 nm (Figure 3B). Charged amino acids on the BAR surface, which have been shown to be crucial for membrane binding, are conserved between dAmph and hBin2 and the electrostatic potentials on their molecular surfaces are also similar (Figure S3).

The prototypical N-terminal amphipathic helix (called helix zero, H0, because the first helix observed in the BAR structure is called helix 1) is that of rEndoA1 where the first 16 residues have clear amphipathic characteristics and fold on membrane binding [4]. A 24 and 28 amino acid long helix, H0, would be possible for dAmph and Bin2, respectively (Figure 3C). Deletion of the predicted amphipathic helix of hBin2 resulted in the loss of a stable membrane interaction (Figure 3D). hBin2 helix H0 also appeared to be more amphipathic than that in rEndoA1 and dAmph owing to the specific distribution of charged and hydrophobic amino acids on its two faces. One of the most striking differences in helix H0 sequence is the presence of bulky hydrophobic residues (F13 and F21) in hBin2. In dAmph these residues are less hydrophobic (Figure 3D). We mutated F13 and F21 to alanines in hBin2 N-BAR to better understand the determinants of hBin2’s enhanced affinity for membranes (Figure 3D). This greatly inhibited membrane binding, highlighting their importance in this critical interaction interface. The stronger affinity of hBin2 over dAmph for membranes and the large hydrophobic residues on the amphipathic helix may also influence the stable curvature observed (not investigated further).

The ability of hBin2 to deform membranes was studied *in vivo* by transfecting hBin2-EGFP (the tag was located at the C-terminus of the protein) in COS-7 cells (where the protein is not normally expressed) (Figure S4A). Tubules were readily visible but it is worth noting that expression of an N-terminal tagged protein (myc-tag) was largely cytosolic, although some tubules were observed (Figure S4B). Since this tag is acidic and adjacent to the predicted amphipathic helix, it may be that the negatively charged tag did not permit helix H0 to correctly fold and insert into the negatively charged membrane. A similar observation for amphiphysin, where an N-terminal tag on the amphipathic helix of amphiphysin also prevented tubule formation when the protein was overexpressed in cells [2].

### Bin2 is Targeted to Podosomes in Adherent Leucocytes

**Figure 4:**
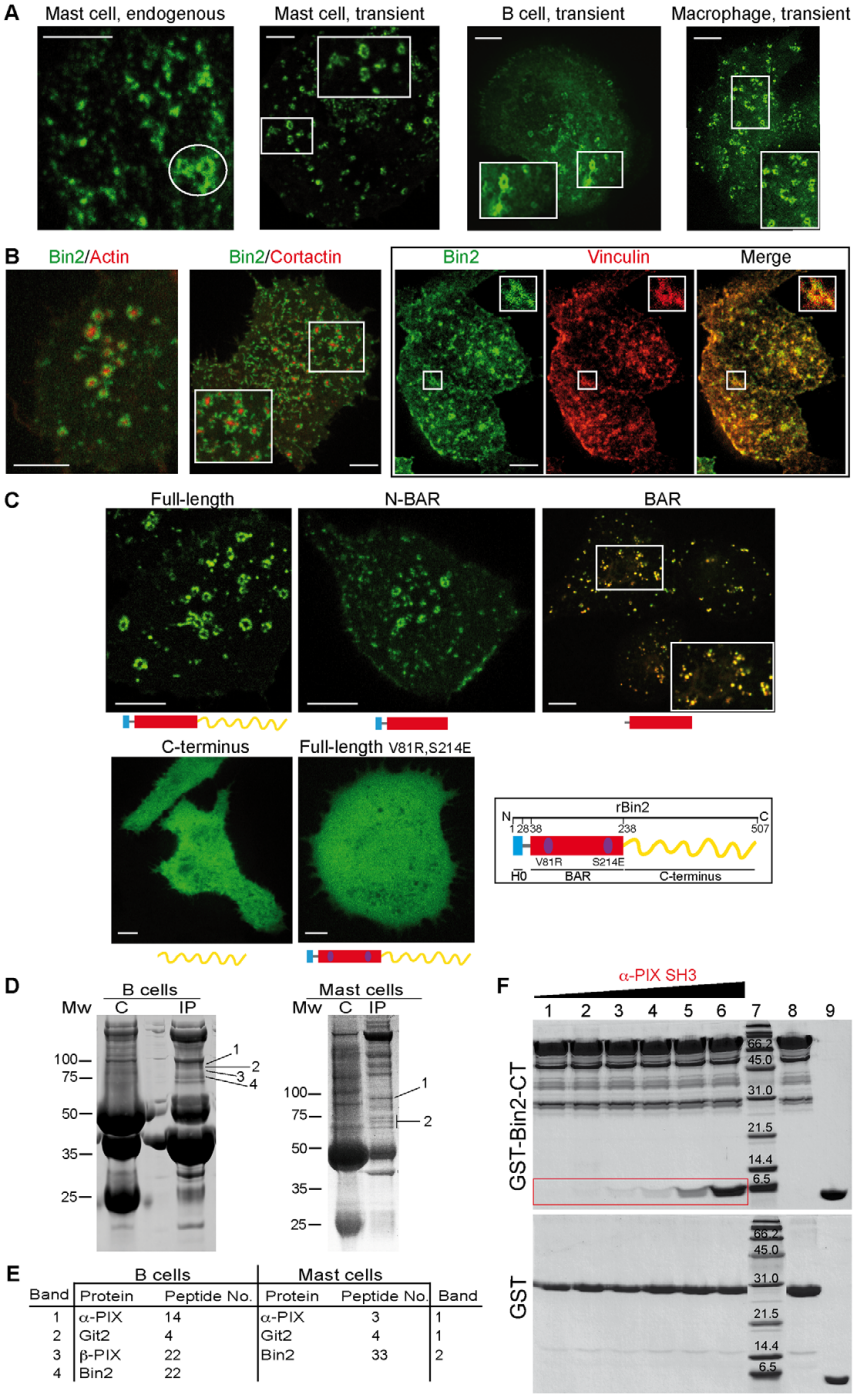
Bin2 localizes at podosomes of adherent leucocytes and is an adaptor for SH3-domain containing proteins. *A* : Epifluorescence images of endogenous Bin2, stained with a polyclonal anti-hBin2 (BACT) antibody, in rat mast cells (RBL·2H3 cells). Transiently overexpressed Bin2-EGFP in mast cells (live cell imaging), human B cells (fixed 721.221 cells), and mouse macrophages (BAC1·2F5 cells, live cell imaging). Podosome like structures are highlighted by the white circle. Boxed areas show enlarged regions. Scale bars, 5µm. ***B***: Bin2 and podosome markers: snapshots of living RBL cells transiently expressing rBin2-EGFP and LifeAct-mCherry or cortactin-mCherry. Box on the right shows fixed RBL·2H3 cells expressing rBin2-EGFP (left) stained with an anti-Vinculin Ab (middle). Small panels show maximized views of highlighted areas (white boxes). Scale bars, 5µm. ***C***: A dimeric N-BAR domain is required for Bin2 targeting to podosomes. Snapshots of living rBRAP-siRNA treated RBL·2H3 cells transiently expressing different rBin2-EGFP siRNA-insensitive mutants: full-length protein, the N-BAR only (1–238), which also localizes at these adhesive structures, the BAR domain (32–238), which goes to lysosomes (the image shows colocalization with lysotracker (red), the C-terminal end (239-end), which is cytosolic and the full-length dimerization mutant (V81R, S214E), mutations destabilize dimer formation and the protein becomes cytosolic. Scale bars, 5 µm. ***D***: Bin2 immunoprecipitations (IPs) from human B cells (721.221) (left) or rat mast cells (RBL·2H3) (right) using our BACT Ab shows binding with PIX and Git2 proteins. In control experiments (labelled as C) these proteins are missing. ***E***: LC-MS/MS data for the bands indicated in (D). ***F***: Bin2 directly interacts with α-PIX. In vitro pull-down assays using GST-hBin2-CT versus a GST (control) with purified human α-PIX SH3 domain. Lanes 1–6 show the GST pull-downs. The amount of α-PIX increases over these samples and only binds to GST-Bin2-CT (red box). Lane 7, broad range molecular markers, BioRad. Molecular weights (kDa) are indicated. Lane 8 GST-hBin2-CT or GST alone and lane 9 α-PIX alone. Proteins were detected by Coomassie stain.

Bin2 was found to be endogenously expressed in mast cells (RBL·2H3 cell line), B cells and macrophages (Figure 1 and data not shown). Both endogenous and overexpressed Bin2-EGFP localized to ring-like structures around the actin core of podosomes (Figure 4A, B). After silencing endogenous Bin2 using siRNA, podosomes were still observed but the dynamics and numbers changed (see below). An siRNA-insensitive Bin2-EGFP construct overexpressed in siRNA treated cells localised to podosomes (Figure 4C and Movie S1; similar to Bin2 over-expression in non-silenced cells). A similar siRNA-insensitive mutant which contained only the N-BAR domain (amino acids 1–238) was also found at podosomes (Figure 4C and Movie S2). In contrast, Bin2 C-terminus (amino acids 238 to end) was cytosolic (Figure 4C). Removal of the N-terminal amphipathic helix (Bin2 BAR domain, amino acids 32–238) resulted in a lysosomal localization (Figure 4C). Thus the N-BAR domain is sufficient for targeting to podosomes.

To verify the importance of dimerization on membrane targeting we mutated two conserved amino acids (V81R and S214E) that participate in dimer formation/stability (Figure 2D). Both regions in the structure would not be able to accommodate long side-chain amino acids. Comparative analysis of WT and mutant proteins by gel filtration suggested that the dimerization properties are indeed reduced (but not abolished) by the mutations (Figure S5). *In vivo*, Bin2 V81R,S214E was cytosolic, demonstrating that proper dimer formation is essential for membrane binding (Figure 4C). Taken together these data suggest that an intact N-BAR dimer is both necessary and sufficient for targeting of Bin2 to podosomal membranes.

Podosomes are known to be rich in phosphoinositides (PIP), and PI3K signalling is necessary for podosome formation [10], [11], [12]. Consistent with its localisation to podosomes, we found that Bin2 had a higher affinity for PIP-enriched membranes in lipid cosedimentation assays, although there was no clear preference for a particular PIP (Figure 3E).

### Bin2 is an Adaptor for SH3-domain Containing Proteins at Podosomes

Using B or mast cell protein extracts and an antibody raised against hBin2, we were able to coimmunoprecipitate Bin2 together with α-PIX, β-PIX and Git2 (Figure 4D, E). It is known that PIX and Git (which bind paxillin) constitute a complex that is located at the ring-like structure of podosomes [13], like we find for Bin2. While α-PIX is enriched in haematopoietic cells (like Bin2), β-PIX is more widely expressed [14].

Bin2 contains several polyproline regions, which usually mediate interactions with SH3 domains. hBin2 C-terminus (Bin2-CT) contains two consensus PxxP sequences (PTSPR at positions 445–449 and PEKPVR at positions 472–477), as well as some others that do not follow any known consensus sequence. Given that PIX contains a SH3 domain and coimmunoprecipitates with Bin2 (Figure 4D, E) and both proteins are located at podosomal ring-like structures, we investigated if both proteins physically interact using *in vitro* pull-down experiments (Figure 4F). α-PIX SH3 domain directly bound to GST-Bin2-CT but not to GST alone. .

To gain further insight into the molecular function of the Bin2 C-terminus, pull-down assays were performed using GST-tagged hBin2-CT and protein extract from B lymphocytes (721.221), macrophages (BAC1·2F5) and mast (RBL·2H3) cells (Figure S6A and data not shown), all of which show high levels of endogenous Bin2 expression (Figure 1B). The N-BAR protein Endophilin A2 was identified as a Bin2 interacting partner by LC-MS/MS of Coomassie stained bands and by western blots (Figure S6A). EndoA1 was pulled-down from brain extracts and Endophilin A3 from testis (Figure S6A). EndoA2 is in general ubiquitously expressed and it has been previously shown that EndoA2 is located at podosome-like adhesions [15], [16], although its function here is not understood. When rBin2 and rEndoA2 are transiently co-expressed in mast cells, both proteins colocalized at the ring-like structure of podosomes (Figure S6C). Bin2 C-terminus interacts directly with Endophilin (Figure S6B). Taking into account the similarities between the N-BAR of Bin2 and Endophilin, we performed pull-down experiments to determine if the proteins can also interact through N-BAR heterodimerization. We did not observe an association (Figure S6B). The interaction of Bin2 with all EndoA isoforms is consistent with the strong similarity between the SH3 domains of Endophilin A1, 2 and 3.

Taken together, these data suggest that Bin2 C-terminus functions as an adaptor for SH3-domain-containing proteins at podosomes, including PIX proteins, which are regulators of podosome function [13], [17] and Endophilin A2, whose function at podosomes remains unknown.

### Bin2 Regulates Cell Movement

To further investigate Bin2 function we examined podosome number in cells treated with Bin2 siRNA. Interestingly, we observed a reduction in podosome density in Bin2-depleted cells suggesting a role for Bin2 in podosomal formation (Figure S7A,B). Furthermore, the persistence time of remaining podosomes was greatly prolonged (reflecting more stable adhesions). This suggests a role for Bin2 in podosomal assembly and disassembly (Figure S7C, D).

**Figure 5:**
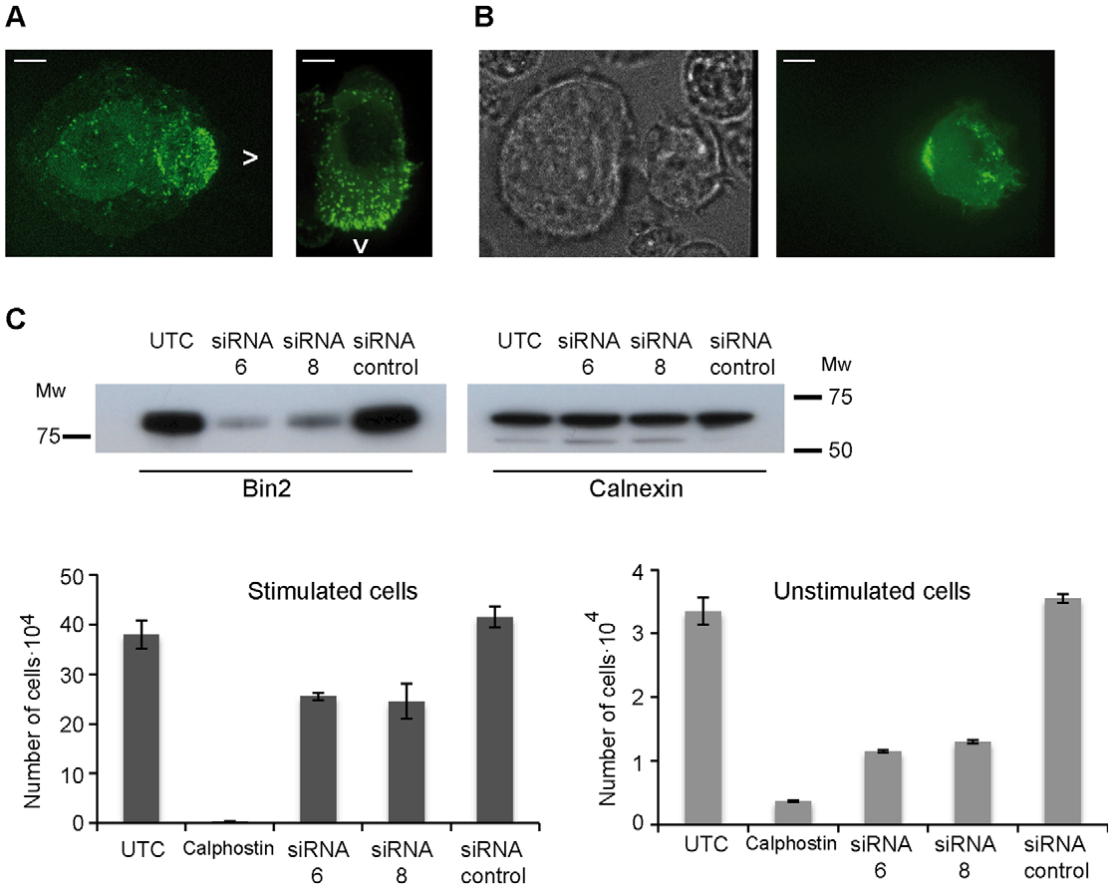
Bin2 regulates cell movement. *A* : Bin2 is at the leading edge of moving cells: (A) Snapshots of a migratory macrophage (mouse BAC1·2F5 cell line, left) and a B cell (human 721.221 cell line, right) overexpressing Bin2-EGFP. Arrows indicate the direction cells are moving. Scale bars, 5 µm. ***B***: Polarization of hBin2-EGFP to the leading edge of a B cell (human 721.221 cell line) that is migrating towards a neighbouring NK cell (human YT cell line). Phase contrast image (left) and epipluorescence micrograph (right). Scale bars, 5 µm. ***C***: Transwell migration assay with monocytes (human U937 cell line). Top: Western blot showing the depletion of Bin2 when cells are treated with siRNA6 and siRNA8 (Dharmacon). Calnexin was used as a loading control. Bottom: Bin2-depleted monocytes (siRNA6 and siRNA8) show a decreased motility when compared with untransfected cells (UTC) or cells transfected with a control siRNA. Cell migration was performed under stimulating (addition of 60 nM RANTES, left) and non-stimulating (right) conditions. An inhibition of cell movement (addition of 5 µM calphostin, a PKC inhibitor) was also performed as a control. Data are the mean ± SD.

The Git-PAK-PIX complex has been found at the leading edge of migratory cells (reviewed in [13]) and has been implicated in neutrophil direction sensing [18]. To determine if Bin2, which interacts with PIX, is also found at the leading edge, we stimulated macrophages with the chemokine CSF-1 and studied Bin2 localization. Bin2 localized to the leading edge of migratory macrophages and B cells (Figure 5A). We also observed similar localization of Bin2 in B cells, when moving towards NK cells (Figure 5B).

Given the role of podosomes and the leading edge in cell migration, we conducted migration assays using a modified Boyden’s chamber assay. Movement of two different leucocytic cell lines was examined: monocytes (Figure 5C) and mast cells (Figure S8). In both cases, the depletion of Bin2 reduced cell movement. The reduction in migration was most marked in resting monocytes exposed to a chemokine gradient where a 60% inhibition in migration was observed. From these data we have no explanation for the difference in movement between stimulated and unstimulated cells, but the stimulated cells are much more motile.

### Bin2 and Its Affects on Phagocytosis

**Figure 6:**
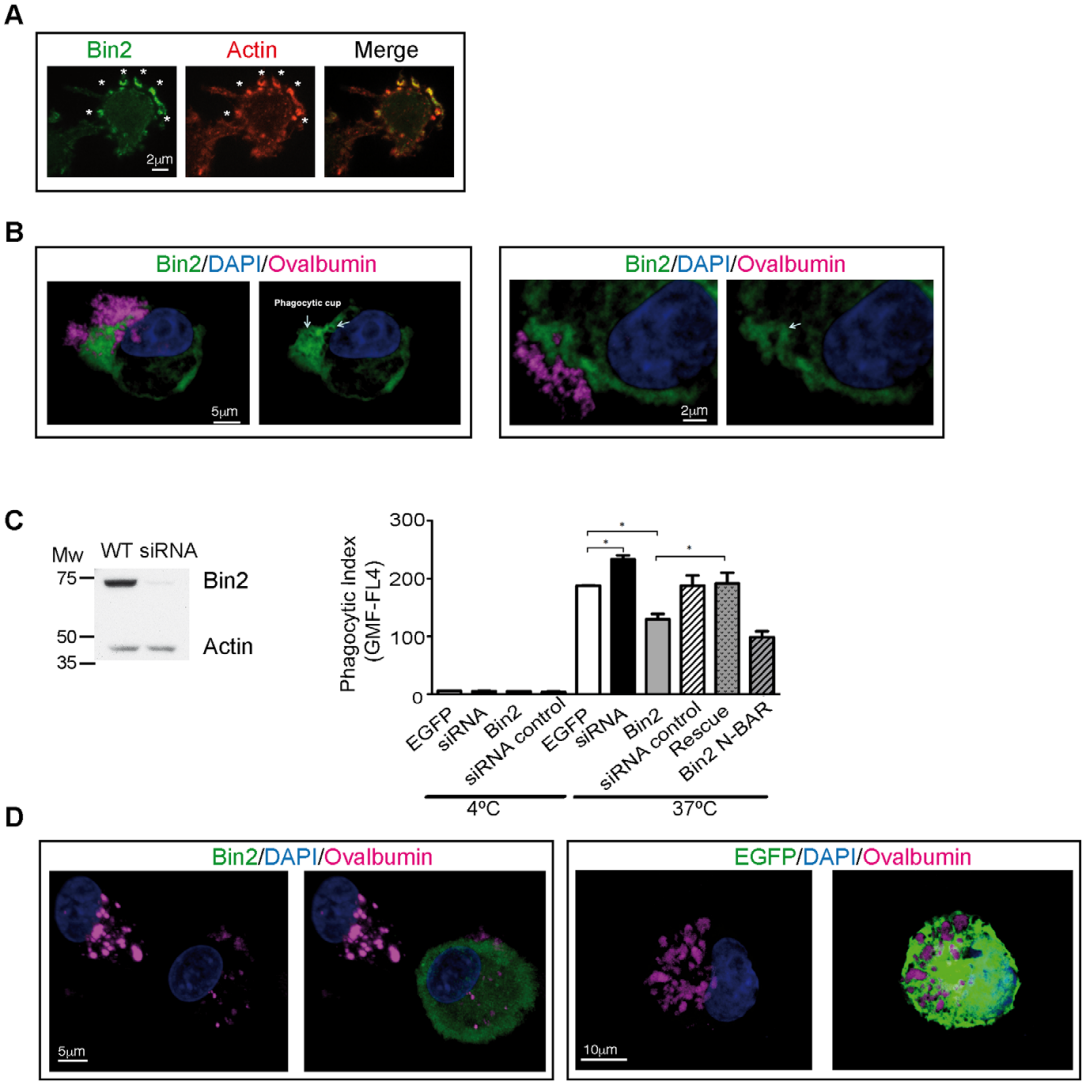
Bin2 is located at the phagocytic cup of macrophages and regulates phagocytosis. *A* : Epifluorescence micrographs of fixed macrophages (mouse BAC1·2F5 cell line) transiently overexpressiong Bin2-EGFP and LifeAct-mCherry. Cells were incubated with 0.9 µm latex beads for 3 min. before fixation to be able to visualize phagocytic cups (white stars). ***B***: Confocal micrographs of two different rat alveolar macrophages (RAM) over-expressing Bin2-EGFP. Cells were incubated with alexa-labelled immune-complexed ovalbumin (pink) for 10 minutes at 37°C. Nuclei stained with DAPI (blue). Bin2 is enriched at the phagocytic cup. ***C***: Left: western blot of RAM lysates from wild type (WT) and 48h-rBin2 siRNA transfected cells developed with polyclonal anti-hBin2 (BACT) and anti-actin (loading control) antibodies. Molecular weight markers (Broad Range, Promega) are indicated. Right: phagocytosis assay (expressed as phagocytic index (geometric mean fluorescence of positive cells)) performed with RAM cells at 4° (surface binding without internalization) and 37°C (internalization). Cells were incubated with alexa-labelled ovalbumin for 45 min before analysis by flow cytometry. Uptake from cells overexpressing rBin2-EGFP (Bin2) and rBin2 N-BAR-EGFP (N-BAR) and Bin2-depleted cells (Bin2 siRNA) where compared with control experiment (EGFP). Overexpression of a siRNA-insensitive protein on Bin2 depleted cells rescues the phenotype (Rescue). A siRNA control was also performed (siRNA control). Data are the mean ± SD. Significance was calculated using the Student’s t test (* = p<0.05). ***D***: Confocal micrographs of RAM cells over-expressing Bin2-EGFP (Bin2, green, left) or control (EGFP, green, right). Cells were incubated with alexa-labelled immune-complexed ovalbumin (pink) for 120 minutes 37°C. Nuclei stained with DAPI (blue).

Since adhesion, motility and phagocytosis are all actin-based activities, we investigated the effects of Bin2 on phagocytosis. We imaged the formation of phagocytic cups in macrophages using Bin2-EGFP transient expression. After incubation of macrophages with 0.9 µm latex beads, Bin2 was found to clearly colocalize with F-actin at phagocytic cups (Figure 6A).

Phagocytosis was examined using the uptake of fluorescent ovalbumin or heat-killed *Streptococcus pneumonia* by rat alveolar macrophages at different time points at 4°C (to assess surface binding without internalization) and 37°C (to assess internalisation). We either over-expressed different constructs of rBin2 or silenced the endogenous protein (Figure 6B, C, D and Figure S9). Bin2 knockdown increased phagocytosis, while the over-expression of Bin2 inhibited particle uptake. Over-expression of the N-BAR produced a similar effect to the full-length construct. The over-expression of an siRNA-insensitive construct on Bin2 depleted cells rescued the observed phenotype. These data suggest that Bin2 inhibits phagocytosis.

## Discussion

In this study we have shown that Bin2 has an N-terminal N-BAR domain that can direct the protein to features on the plasma membrane and can affect membrane curvature on liposomes *in vitro*. Given the similarity of Bin2 N-BAR domain to the N-BAR domains of Amphiphysin and Endophilin A proteins, one might have assumed that this protein will have a major role in endocytosis. However it does not localize to sites of clathrin-mediated endocytosis (data not shown) and indeed it does not have a C-terminal SH3 domain like that found in Amphiphysin and Endophilin A proteins. This does not preclude it is playing a role in endocytosis, given that we have shown it can interact with Endophilin *in vitro* and in cells, but the absence of an SH3 domain means that it does not interact with Dynamin or Synaptojanin, or with N-Wasp. Instead the C-terminus interacts with α-PIX and Git2, which are found at sites of actin based membrane remodelling, such as podosomes and the leading edge regions of motile cells. We found that Endophilin A2 still localized at podosomes in Bin2 depleted mast cells. It is also known that the PIX-Git complex localizes at the ring of podosomes through the binding of Git to paxillin, which also binds integrins [14]. This suggests that localization of Endophilin A2 and PIX does not depend on Bin2 but it might be regulating their function by permitting or impeding the binding of other proteins through their SH3 domains. Further work will be necessary to elucidate the mechanism.

Podosomes are abundant in monocytes, macrophages and dendritic cells where we find Bin2 expression and podosomes act as dynamic foci of cell-matrix adhesion and of matrix remodelling. Structurally, they have a central core of F-actin (perpendicular to the cell surface), with a central membrane invagination where dynamin is located. The core contains actin-associated proteins and is surrounded by a ring-like structure containing adhesion proteins, including actinin, paxillin, talin and vinculin, cytoskeletal proteins, such as myosin II and tropomyosin, and other regulatory proteins including PIX and Git proteins [13], [19], [20].

Cell migration requires that cells interact with their environment through dynamic contact with the extracellular matrix (ECM) or with other cells. In most cell-types, integrin-based adhesions are organized into short-lived focal complexes. For highly migratory cells like macrophages, these interactions also take the form of podosomes. The initial response of an isolated cell to a migration stimulus such a chemokine is to polarise and extend protrusions that come to define the direction of locomotion. It has been proposed that podosomes might provide local anchorage and thus stabilise cellular protrusions, which might enable more efficient direction of migration. [10], [13], [19], [21], [22].

The lack of dynamics and the reduction of podosomes numbers would explain why the silencing of Bin2 inhibits cell migration. Consistent with our observation, the depletion of PAK4 (a PIX interacting protein in podosomes) in macrophages alters podosome formation [17]. Also consistent is the observation that over-expression of PAK1 or ß-PIX in smooth muscle cells triggers podosomes formation [23]. Members of the PAK family are major effectors of the Rho GTPases CDC42 and Rac (reviewed in [24]) and it has been shown that PAK induces reorganization of the actin cytoskeleton by influencing cofilin activity or by phosphorylating cortactin [13]. It has been proposed that the interaction of PAK, PIX and Git would form an interface between adhesion-associated proteins of the ring and the regulation of actin dynamics in the core [13]. Here we show that Bin2 by interacting with PIX might be coupling cell adhesion and actin polymerization with membrane deformation, which must be very tightly coupled events in cell migration. Further investigations need to be carried out to understand the interplay of these proteins for podosome regulation and cell movement.

Monocyte-derived cells such as macrophages and dendritic cells rely on actin-based membrane structures to mediate many of their functions, including chemotaxis and antigen internalisation. In chemotaxis, podosomes are utilised to anchor cells to the ECM and to localise metalloprotease secretion, both of which facilitate migration, whereas phagocytosis, requires the generation of phagocytic cups, to allow the internalisation of foreign material such as bacteria. Some data suggest that podosome formation and antigen internalisation are reciprocal processes; following ingestion of antigen, dendritic cells divert their actin cytoskeleton away from the phagocytic cup and towards podosome formation and cell migration [25]. This would facilitate the designated function of dendritic cells to sample antigens, prior to migration to draining lymph nodes where they may initiate an immune adaptive immune response. Bin2 appears to enhance podosome formation and migration and inhibit antigen internalisation and might therefore be an important molecule in mediating this switch in phenotype. While little is known about direct roles for GIT and PIX proteins during phagocytosis, our study suggests that they may play a role here and it would be interesting for future studies to address this. In terms of a switch between podosome-based adhesion and phagocytosis, we suggest that (while Bin2 appears to have direct roles in both processes) membrane tension together with other specialist proteins (as there are likely to be proteins needed for identity purposes or for the specific membrane curvature involved) may provide the ultimate permissive link for dual regulation of these processes. We suggest that Bin2-mediated membrane dynamics may play a role here, where loss of Bin2, through reduced cell-matrix adhesion, allows sufficient global membrane laxity for phagocytosis to then begin and proceed more easily. There is likely a balance/competition between cell-matrix adhesion and phagocytosis for the development of strong plasma membrane-cytoskeleton connections.

In conclusion, Bin2 is a membrane remodelling adaptor for SH3 domain containing proteins located at dynamic actin-remodelling adhesive structures of immune cells.

## Materials and Methods

### Expression, Purification and Crystallization of Bin2 BAR Domain

Human Bin2 N-BAR domain (1–238) was cloned into pGEX-4T-2 (GE Healthcare). GST-hBin2 N-BAR was expressed overnight at 25°C, in *E. coli* strain DH5α in 2×TY medium. The expression was induced with 0.4 mM IPTG. The N-BAR domain was purified in three steps. Firstly, the cell pellet was resuspended in buffer containing 20 mM HEPES, 500 mM NaCl pH 7.5, 2 mM DTT. Cells were disrupted and the clarified supernatant was incubated with glutathione sepharose 4B beads (GE Healthcare) at 4°C. After several washes, the GST tag was cleaved with thrombin protease (GE Healthcare) in buffer containing 20 mM HEPES, 200 mM NaCl pH 7.5, 2 mM DTT (buffer A) and then loaded onto an ion exchange HiTrap Q FF column (5 ml) (GE Healthcare) to remove contaminating DNA. The protein, which eluted in the flow-through was concentrated and applied to a gel filtration column where it eluted in a single peak. The elution volume was consistent with a dimeric Bin2 N-BAR domain. The far CD spectrum showed that it was predominantly α-helical. During the cleavage of the GST tag the protein was partially degraded (Figure 3A). N-terminal sequencing showed that the N-terminal amphipathic helix was partially cleaved. This likely occurs due to the unfolded nature of this helix in solution [4]. Despite the protein heterogeneity observed, this sample was concentrated to 10 mg/ml for crystallization trials. Crystals of N-BAR 1–238 did not diffract beyond 5 Å resolution. Crystals were solubilised and checked by SDS-PAGE electrophoresis, and were found to be the mixture of full-length N-BAR and the 19 amino acid truncation. Thus we proceeded with an N-terminal truncation.

Human Bin2 BAR domain (residues 20–238; Bin2 N-BAR Δ19), partially lacking the N-terminal amphipathic helix, was cloned into BamH1/Xho1 sites of pGEX-4T-2 (GE Healthcare). The purification protocol was the same as for the native protein. Crystals of Bin2 N-BAR Δ19 were grown by hanging-drop vapour-diffusion techniques in 10% MPD, 7% PEG4000 and 0.1M sodium citrate pH 5.3 at 20°C. Crystals were cryoprotected by increasing the MPD concentration to 25%, mounted in a fiber loop and cryocooled in liquid nitrogen for data collection. Crystals from the seleno-methionine (Se-Met)-substituted protein were grown in similar conditions. The purification protocol for the Se-Met derivative was the same as for the native protein. In this case, the DTT concentration was raised to 5 mM to avoid Se oxidation. Mass spectrometry analysis showed that four selenium atoms were incorporated.

Drosophila Amphiphysin (dAmph) (1–244) and rBin2 (1–238) N-BAR domains were cloned into the Nde1/Not1 and BamH1/Xho1 sites, respectively, of pGEX-4T-2 (GE Healthcare). The purification protocol for rBin2 and dAmph constructs was identical to the one described above for the Bin2-NTΔ19 construct.

### Diffraction Data Collection and Structure Solution

Crystals were initially indexed in point group P422. Different data sets were collected on beamline ID29 at the ESRF, Grenoble. A fluorescence scan was performed with a Se-Met crystal (Se-Met 2) to collect a MAD data set. In this case, 180° were collected at the peak energy, then 90° at the inflexion point followed by another 90° at a remote high-energy point, in that order. The initial peak dataset was recollected at the end to analyze if there was radiation damage. Using another Se-Met crystal (Se-Met 2), a high multiplicity (360°) SAD data set was collected. Finally, a native data set was collected (a 180° wedge) since these crystals diffracted at higher resolution. The crystallographic data were processed using Mosflm [26] and scaled with Scala [27]. During the analysis of the data we realized that the crystals suffered pseudo-merohedral twinning and that the true space group was P2_1_2_1_2_1_, with the twinning operator k,h,-l. See Table 1 for further details about the data processing.

Using the MAD data, eight Se sites were found using Sharp [28]. The MAD phases were not good enough to yield a good experimental electron density map. Then, we added the anomalous data coming from the SAD data set and the isomorphous information coming from the native data. This new set of phases was improved by solvent flattening with Solomon [29] using a solvent content of 57.5%. Twinning was ignored during the phasing. The resulting experimental map was good enough to identify the position of two molecules in the asymmetric unit. The calculation of the anomalous difference map permitted the location of the four Se in each molecule, which greatly aided model building.

The structure was refined against the native data using Refmac [30] and it was necessary to consider the twinning at this stage (Refmac twin fraction 0.11). During the refinement the model was updated using Coot [31]. The final model included residues 37 to 238, that is, the N-terminal amphipathic helix was disordered in both subunits. The structure quality was analyzed with MolProbity [32]. The Ramachandran plot showed that the 95.4% of the residues were in most favoured regions and 4.7% in additional allowed regions. Ribbon figures were drawn and electrostatic potentials were calculated with CCP4mg [33]. The crystal structure and structure factors were deposited in the Protein Data Bank (PDB code: 4I1Q).

### Analytical Ultracentrifugation

Equilibrium sedimentation experiments with hBin2 N-BAR were done in the Beckman Optima-XLI instrument at 20°C, using 50Ti rotor and 6-sector centrepieces, at 230 and 280 nm at 20,000, 30,000 and 40,000 rpm. Data were analysed using the UltraSpin software (D. Veprintsev). Partial specific volume was calculated from the protein sequence to be 0.7341 using Sedenterp software (J. Philo). Molecular mass of a monomer was 25996, and extinction coefficient at 280 nm at 29890M^−1^cm^−1^. Extinction coefficient at 230 nm was estimated from the ratio of 230/280 nm to be 141000M^−1^cm^−1^. Buffer conditions were 20 mM Tris, 200mM NaCl, 0.1 mM DTT.

### Liposome Cosedimentation Assays

Liposomes were prepared from total brain lipids (Folch fraction 1, Sigma) or from synthetic lipids (Avanti Polar Lipids). Synthetic lipids were prepared by mixing 30% PS, 10% cholesterol, 59% PC and 1% PIPs, which were previously subjected to protonation. Once desiccated in a non-oxidizing atmosphere, lipids were hydrated in buffer A, to a final concentration of 1 mg/ml. The sample was sonicated for 20 seconds. The liposomes were subsequently passed through 0.8 µm Nucleopore polycarbonate filters (Whatman) 9 times by syringe extrusion.

hBin2 and dAmph (1–244) N-BARs were expressed and purified using the same protocol as the one described before.

Each protein (at 7 µM, final concentration) was incubated for 30 minutes with 0.5 mg/ml liposomes. The solution was then centrifuged at 65000 rpm in a TLA100 rotor (Beckman) for 15 min. Supernatant and pellet fraction were separated and resuspended in equal volumes of reducing sample buffer and run on 4–12% Bis-Tris SDS-PAGE gels.

### Negative-stain Electron Microscopy

dAmph and hBin2 N-BAR domains (5 µM final concentration) were incubated with 0.6 mg/ml liposomes (Folch fraction 1) for 15 min at room temperature. Glow discharged carbon-coated copper grids (CANEMCO) were loaded for 30 s in the protein-liposome mixture. The grids were then stained with 2% uranyl acetate. Excess solution was removed by touching the edge of the grids with filter paper. The grids were visualized using a Philips transmission electron microscope.

### Immunoprecipitations

Human B cell (721.221) and rat mast cell (RBL·2H3) protein lysates were generated by incubating cells in buffer (20 mM HEPES, 150 mM NaCl, 1 mM DTT, 0.3% Triton X-100, 1% NP-40 and protease inhibitors) for 40 minutes at 4°C. Disrupted cells were centrifuged at 50,000 rpm in a TLA100·4 for 30 minutes at 4°C. The supernatant was removed and added to 50∶50 mix of protein A:G Sepharose beads (Amersham Biosciences), to which anti-human Bin2 polyclonal antibody (BACT, more details in the Antibody section) had been previously bound (at 4°C for 4 h). The mixture was incubated at 4°C for 3 hours. Beads were washed three times with the initial buffer and then once in buffer without detergent, before electrophoresis by SDS-PAGE and Coomassie staining. Protein bands of interest were analyzed by mass spectrometry using LC-MS/MS. Control experiments were performed with pre-immunized rabbit serum instead of the BACT antibody.

### LC-MS/MS Spectrometry

Peptides from in-gel trypsin-digested protein bands were separated by liquid chromatography on a reverse phase c18 column. The eluted samples were introduced directly onto a Q-STAR hybrid tandem mass spectrometer (MDS Sciex). The spectra were searched against a NCBI non-redundant database with Mascot MS/MS ion search.

### Pull-down Assays with Rat Tissue and Leucocyte Protein Lysates

The C-terminal region of hBin2 (amino acids 237-565) (Bin2-CT) was cloned into Nde1/Not1 sites of pGEX-4T-2. The protein was overexpressed overnight at 25°C in *E. coli* strain DH5α in LB medium. Expression was induced with 0.1 mM IPTG. The cell pellet was resuspended in buffer comprising 20 mM HEPES pH 7.5, 500 mM NaCl, 10 mM EDTA, 1 mM DTT, 0.1 mM PMSF plus protease inhibitors (1∶1000 dilution of Cocktail Set III, Calbiochem) and DNAse (HT Biotechnology). GST protein was also expressed and used as a control. After cell disruption and centrifugation, the supernatant was incubated with glutathione sepharose 4B beads (GE Healthcare) at 4°C. The beads were extensively washed and GST-Bin2-CT was eluted in the same buffer supplemented with 20 mM glutathione (Calbiochem). The protein sample was dialyzed overnight against buffer 20 mM HEPES pH 7.5, 150 mM NaCl, 10 mM EDTA, 5 mM DTT, plus protease inhibitors (1∶1000 dilution of Cocktail Set III, Calbiochem) (buffer H). The final protein showed a degradation pattern that suggested poor folding (in accordance with the high number of prolines).

Rat brain and testis were homogenized in buffer H plus 0.1%Triton X-100 (BDH Laboratories Supplies). B lymphocytes (721.221 cell line), macrophages (BAC1·2F5 cell line) and mast cells (RBL·2H3 cell line) were centrifuged and homogenized in buffer H plus 0.1% Triton X-100 and 1% NP40 (BDH Laboratories Supplies) and centrifuged at 4°C for 10 min at 60000 rpm in a TLA100.4 rotor (Beckman). The supernatants containing the cytosolic proteins were kept on ice.

For pull-down experiments, the protein extracts from these cells/tissues were incubated with GST-Bin2-CT immobilized on glutathione-sepharose beads (GE Healthcare) at 4°C for 40min. A control experiment was also performed using only GST protein. The beads were washed twice with 40 volumes of buffer H plus the corresponding detergents, followed by a wash without detergents. Samples were analysed by Coomassie-staining following 4–12% Bis-Tris SDS-PAGE. Additional bands that were present in the experimental lane with respect to the control (GST only) were excised and analyzed by LC-MS/MS spectrometry. Analysis of additional bands was also performed by western blot, using rabbit anti-Endophilin1 and 2 antibodies (ZYMED Laboratories, Invitrogen respectively).

Animal work was conducted under Animals (Scientific Procedures) Act 1986 and subject to local ethical approval by MRC Ethical Review Committee.

### Pull-down Assays using Overexpressed and Purified Proteins

Human α-PIX SH3 domain (amino acids 160–222) (cloned into pGEX-4T-2, GE Healthcare) was overexpressed in Rosetta2 bacteria (Novagene) and affinity-purified in 20 mM HEPES pH 7.5, 300 mM NaCl, 1 mM DTT plus protease inhibitors (Roche). Overnight on-column cleavage of the GST tag was performed using thrombin protease at 4°C. The full-length rat EndophilinA1 (cloned into pGEX-6P-2, GE Healthcare) was over-expressed in BL21(DE3) pLysS and affinity-purified in 20 mM HEPES pH 7.5, 300 mM NaCl, 1 mM EDTA, 0.1 mM PMSF, 2 mM DTT plus protease inhibitors (1∶1000 dilution of Cocktail Set III, Calbiochem). Overnight on-column cleavage of the GST tag was performed using PreScission protease at 4°C. Finally a gel filtration was performed in 20 mM HEPES pH 7.5, 150 mM NaCl, 1 mM DTT (buffer B). All constructs were dialyzed overnight against buffer B.

Pull-down experiments were performed in buffer B plus 0.1% Triton X-100 to avoid nonspecific binding. GST-hBin2-CT or GST-hBin2 N-BAR (10 µg) were incubated on glutathione beads with different amounts of human α-PIX SH3 domain (10, 25, 35, 50, 75 and 100 µg) or rat Endophilin A1 (2.5, 5, 10, 25 and 50 µg) for 60 min. The beads were then washed twice with 40 volumes of buffer B plus 0.1% Triton X-100, followed by a final wash without detergents. The samples were subjected to SDS-PAGE electrophoresis.

### Cell Culture, Transfections and siRNA

Human B lymphocytes (cell line 721.221, ATCC) and T cells (Jurkat, ATCC) (gifts from Hugh Reyburn, Department of Pathology, University of Cambridge) were grown in RPMI 1640 with GlutaMAX (GIBCO) supplemented with 10% foetal bovine serum (FBS). Rat mast cells (RBL·2H3, ATCC) and human natural killer cells (YT, DSMZ), (gifts from Gillian Griffiths, CIMR, Cambridge) were grown in DMEM with GlutaMAX (GIBCO) supplemented with 10% FBS. Rat alveolar macrophages (NR8383, ATCC) were grown in F12K medium supplemented with 15% heat inactivated FBS and 1.5 g/l NaHCO_3_. Mouse macrophages (BAC1·2F5) [34] (a gift from Richard Stanley, Albert Einstein College of Medicine, NY) were grown in MEM-alpha with GlutaMAX (GIBCO) supplemented with 0.02 mg/ml L-Asparagine, 0.05 mM ß-mercaptoethanol and 3000u/ml CSF-1. Human monocytes (cell line U937, ATCC) were cultured in RPMI supplemented with 10% FBS. B and NK cells were transfected using nucleofector technologies, while rat mast cells, rat alveolar macrophages, U937 and mouse macrophages were transfected with a microporator (Digital Bio Technology).

For transient protein expressions the following constructs were used: pEGFP-N3-hBin2, pEGFP-N3-rBin2, pEGFP-N3-rBin2 N-BAR (amino acids 1–238), pEGFP-N3-rBin2-CT (amino acids 237 to end), pEGFP-N3-rBin2-NTΔ31 (amino acids 32–238), pEGFP-N3-rBin2 (V81R, S214E) dimerization mutant, pmCherryN1-rEndophilin A2, pmCherryN1-rCortactin and pmCherryN1-Lifeact (the latter two being gifts of Marcus Taylor, Christien Merrifields laboratory, MRC-LMB, Cambridge). After transfection, cells were plated on fibronectin-coated cover slips to permit cell attachment.

In order to silence Bin2 in rat cells, 10^5^ cells were transfected with 0.3µg rBin2-specific siRNA (siRNA 9) with target sequence GCCGGAAACUGGUCGACUA (ON-TARGETplus J-082046-09, Thermo Scientific). An siRNA control experiment was performed using a siRNA designed for the human protein which did not target rBin2. hBin2 was silenced using the same protocol and the hBin2-specific siRNAs with target sequence GUCGGAAACUCGUGGACUA (SiRNA 6) (ON-TARGETplus J-020919-06, Thermo Scientific) and GAACCCGCACCGCAAGUGA (SiRNA 8) (ON-TARGETplus J-020919-08, Thermo Scientific). siRNA control experiment was performed using a siRNA designed for the rat protein which did not target hBin2.

Bin2-EGFP constructs insensitive to the siRNAs were also made to rescue the phenotype in Bin2-silenced cells (rBin2 siRNA sequence was GCCGGAAACUGGUCGACUA and in order to avoid siRNA targeting, the DNA sequence in that region (bp 428–446) was mutated to GGCGCAAGCTCGTGGATTA).

### Antibodies

Polyclonal antisera were generated against Bin2 (BACT) by immunising rabbits against His-Bin2-CT. BACT Ab was affinity purified in house. Polyclonal BACT, anti-EndoA1 and anti-EndoA2 (Invitrogen), anti-calnexin and anti-actin (Sigma) were used for western blotting analyses. BACT, Alexa-fluor 546 phalloidin, Lysotracker Red (Invitrogen-Molecular Probes) and mouse anti-vinculin monoclonal antibody (clone hVin-1, Abcam) were used to visualize Bin2, F-actin, lysosomes, and vinculin, respectively.

### Immunofluorescence Analysis

Cells plated on fibronectin cover slips were fixed in 3.2% paraformaldehyde in phosphate-buffered saline (PBS) for 15 min at 37°C, then washed and blocked in 5% goat serum, with 0.1% saponin, in PBS before staining with the appropriate antibodies in 1% goat serum, 0.1% saponin in PBS using standard protocols. Epifluorescent micrographs were taken with a Perkin Elmer spinning disk.

### Live-cell Fluorescent Microscopy and Analysis

Cells were grown on MatTek dishes and before imaging the medium was changed to MEM-alpha without phenol red supplemented with 20 mM HEPES pH 7.4 plus 5% FBS and placed into a temperature controlled chamber on the microscope stage with 95% air:5% CO2 and 100% humidity. Live-cell imaging data were acquired using a fully motorized inverted microscope (Eclipse TE-2000, Nikon) equipped with a CSU-X1 spinning disk confocal head (UltraVIEW VoX, Perkin-Elmer, England) using a 60× lens (Plan Apochromat VC, 1.4 NA, Nikon) under control of Volocity 5.0 (Improvision, England). 14-bit digital images were obtained with a cooled EMCCD camera (9100-02, Hamamatsu, Japan). Four 50 mW solid-state lasers (405, 488, 561 and 647 nm; Crystal Laser and Melles Griots) coupled to individual acoustic-optical tunable filter (AOTF) were used as light sources to excite TagBFP, EGFP, mCherry, and Alexa546, as appropriate. Rapid one or two colour time-lapses were acquired at 1s intervals.

### Cell Movement

Macrophages (BAC1·2F5 cell line) transiently overexpressing Bin2-EGFP were washed in CSF-1 free medium, plated out on fibronectin coated cover slips and CSF-1 starved overnight. Drops of medium containing CSF-1 were added to the imaging plate to let the cells move towards the CSF-1 gradient. The basal surface was imaged using a spinning disk. B cells expressing Bin2-EGFP were also imaged while they were moving.

B cells (721.221), transiently overexpressing hBin2-EGFP and NK cells (YT) overexpressing mCherry were washed twice in serum free media and equal amounts of each cell type were incubated for around 10 minutes before plating and imaging with a spinning disk.

### Transwell Cell Migration

U937 cells (ECACC) were transfected with hBin2 siRNA 6 and siRNA 8 (using EGFP as a reporter) and cultured for 48 h. Cell either stimulated with human recombinant 60nM Rantes (Gibco PHC1054) for 2 h or unstimulated were applied at a density of 1×10^6^ cells/200 microL to a 6.5 mm 8 micron pore size polycarbonate membrane (Costar 3422) and left to migrate for 90 min. As an inhibitor of migration 5microM calphostinC (Calbiochem 208725) was used for 90 min before the start of migration. Unlike RBL·2H3 cells which remain adherent on the lower surface of the membrane, U937 cells migrate through the membrane into the reservoir of medium in the well below. Migration was thus assessed by counting the total number of cells in each well.

RBL·2H3 cells were transfected with rBin2 siRNA 9 (using EGFP as a reporter) and cultured for 48 h. Cells were sorted by FACS for GFP expression. 2×10^6^ cells were added to the wells of a Neuroprobe 96 well manifold and allowed to migrate across a polycarbonate filter with 8micron pores for 6 h. Cells were removed from the top surface of filters by scraping and migrating cells on the bottom surface of the filters were fixed in 3.2% paraformaldehyde. Cells were stained in a solution of HCS CellMask (Invitrogen) and the filters was then analysed on a Licor Odyssey scanner at 650 nm. Overexpression of Bin2 has no detectable effect on migration.

### Phagocytosis Assays

Analysis of phagocytic cup formation was performed by incubating adherent BAC1·2F5 macrophages, transiently overexpressing Bin2-EGFP, with 0.9 µm latex beads for 10 min at 37°C. Cells were fixed with 3.2% paraformaldehyde and F-actin was stained with phalloidin.

Phagocytic assays were performed by incubating alveolar rat macrophages with Alexa-labelled ovalbumin (Invitrogen), or antibody opsonised ovalbumin (opsonised with a rabbit anti-OVA antibody (Sigma, UK) for 1hour prior to use) at 4°C (to assess surface binding with no internalization) and 37°C (to assess internalisation). Macrophages were harvested at different time points (as indicated in the figure), washed three times with cold PBS, and phagocytosis was assessed by flow cytometry (FACSCalibur, BD biosciences). Analysis was performed using FlowJo software (Tree Star, Inc.) to identify transfected cells, and assessing the phagocytic index (geometric mean fluorescence of positive cells) in transfected cells. Some cells were placed on coverslips, fixed in 1% paraformaldehyde and the phagocytic cup or phagocytosis imaged on a Zeiss 710 confocal microscope following nuclear staining with DAPI (Vectashield mounting medium with DAPI, Vector Laboratories, CA).

For pneumococcal phagocytosis assays, *S. pneumoniae* type 14 was cultured to log phase in Todd-Hewitt Broth with 0.5% yeast extract (Oxoid), heat inactivated at 60°C for 1 hour and labelled with ef670 (eBiosciences) as described previously [35]. ef670-labelled *S. pneumoniae* were incubated in PBS or dilutions of heat-inactivated non-immune mouse serum at 37°C for one hour before washing. Aliquots of serum-opsonised and non-opsonised ef670-labelled pneumococci were added to macrophages at 37°C for 60 minutes.

### Podosome Density Analysis in RBL·2H3 Cells

Rat mast (RBL·2H3) cells (10^5^ cells) were transfected with 0.3 µg rBin2-specific siRNA 9 plus 0.2 µg pEGFPN3 or TagBFPN1, (to verify siRNA transfection) using a microporator (Digital Bio Technology). Cells were plated on fibronectin-coated cover slips. After 30 h, cells were detached and subsequently transfected with 0.2 µg pmCherryN1-LifeAct (a marker to visualize F-actin which does not affect actin dynamics) [36]. Cells were plated out and 6h after, the ventral side of cells was imaged every second with a spinning disk to be able to analyze podosomal F-actin columns. A siRNA control experiment was performed using a siRNA designed for the human protein (target sequence GAACCCGCACCGCAAGUGA, ON-TARGETplus, Termo Scientific), which did not target rBin2. We also transfected cells with EGFP as a control. Rescue experiments were performed transfecting rBin2-silenced cells with 0.3 µg of a siRNA-insensitive rBin2-EGFP construct plus 0.2 µg pmCherryN1-LifeAct. Movies were acquired 6h after transfection. The experiments were performed independently with different pulls of cells. Both density and dynamics of podosomes were analyzed. For density calculations, the number of podosomes per cell was counted and divided by the cell basal surface, measured in pixels. When looking at the dynamics, we measured the life span of podosomes: the time since the F-actin column appears until it disappears. We did not take into account in these calculations podosomes that were already formed when the movie started or the ones that did not disappear when the movie finished. Furthermore, to be able to simplify the analysis, we only focused on podosomes that do not perform lateral movements, split or fuse. 290 podosomes were traced to study their dynamics. Three independent experiments with different cell stocks were performed and 20 cells were imaged for quantification.

## Supporting Information

Figure S1 **Bin2 is mainly expressed in leucocyte-enriched tissues.** SDS-PAGE gel showing the amount of loaded protein from cell homogenates of different rat tissues. Right panel shows an antibody blot from spleen extract, showing the antibody detects a single protein species at approximately 70 kDa. The predicted mass of rBin2 is 60 kDa. Molecular weight markers (Broad Range, Promega) are indicated.
(TIF)

Figure S2 **Dimerization properties of hBin2 N-BAR by analytical ultracentrifugation.** Upper panel: Residuals of the direct fit of the data with model describing dimerization equilibrium. Lower panel: Dependence of apparent molecular weight of Bin2 (<M>) on its concentration.
(TIF)

Figure S3 **Electrostatic properties of hBin2,** ***A*** **, and dAmph,** ***B*** **, N-BARs.** Equipotential surface representation contoured at 0.05V. Red and blue stand for negative and positive charges respectively.
(TIF)

Figure S4 **Bin2 tubulates membranes** ***in vivo*** **.** Epifluorescent micrographs of fixed COS-7 cells transiently overexpression hBin2-EGFP, ***A***, and Myc-hBin2, ***B***. Scale bars, 20 µm.
(TIF)

Figure S5 **Gel filtration profiles of WT rBin2 N-BAR (green) vs rBin2 N-BAR V81R,S214E mutant (purple).** Molecular weight standards (BioRad) are indicated with arrows.
(TIF)

Figure S6 **Bin2 interacts with Endophilin A2 at podosomes.** ***A***: Bin2 interacts with Endophilin A (EndoA) proteins. Pull-down assays using lymphocytes (human B cells and mouse macrophages), rat brain and rat testis cytosolic protein extracts. GST-hBin2-CT (238-end) (BCT) was immobilized on glutathione sepharose beads (Input). EndoA2 was pulled-down from B cell and macrophage cell extracts, EndoA1 from brain and Endophilin A3 from testis. The stars show the new bands that appeared after incubation with cellular protein extract (Output). Control experiments with GST (GST) were also performed. Bands were identified by LC-MS/MS and by western blot with the corresponding anti-Pan-EndoA, anti-EndoA1 and anti-EndoA2 Abs (Invitrogen). ***B***: Bin2 interacts directly with EndoA proteins. In vitro pull-down assays with GST/GST-hBin2 (N-BAR and CT) and rEndoA1. In lanes 1–5 increasing amounts of EndoA1 were added to the GST-Bin2 pulldowns (red box). Lane 6, molecular weight markers (Broad range, Promega). Molecular weights (kDa) are indicated. Lane 7, EndoA1 alone. Lane 8, GST-Bin2-CT/NT or GST. ***C***: EndoA2 colocalizes with Bin2 at the ring-like structure of podosomes. Basal surface images of rat mast cells (RBL·2H3) transiently expressing rBin2-EGFP and rEndoA2-mCherry. Scale bar, 5µm.
(TIF)

Figure S7 **Bin2 influences podosome density and lifespan in mast cells (RBL·2H3).** ***A***: Bin2-depleted cells (siRNA) show a decreased density of podosomes with respect to WT (UTC) or siRNA-control cells (siRNA control). Overexpression of a siRNA-insensitive protein in Bin2 depleted cells rescues the phenotype. Light-grey bars show the standard deviation from the mean. ***B***: Basal surface images of live cells. F-actin is labelled by LifeAct-mCherry (red) while Bin2-EGFP is in green. ***C***: Podosomes from Bin2-silenced cells are not dynamic. ***D***: Representative examples of kymographs showing the lifespan of podosomes. The length of the movies is indicated.
(TIF)

Figure S8 **Bin2 regulates mast cell (RBL·2H3) migration.** ***A***: Western blot showing the effective depletion of rBin2 when cells are treated with siRNA. Actin was used as a loading control. Molecular weight markers (Broad Range, Promega) are indicated. ***B***: Transwell migration of cells transfected with siRNA control vs siRNA under stimulating conditions (addition of 20 µg/ml fibronectin). siRNA control cells were also treated with 15% FBS to inhibit cell migration. Amount of migrating cells is expressed as fluorescence units. Data are the mean ± SD. ***C***: Wells showing the amount of cells that were able to migrate during the experiment. Cells were stained with HCS CellMask (Invitrogen) for proper visualization.
(TIF)

Figure S9 **Phagocytosis assays**. ***A***: Levels of Interferon γ (IFN-γ) production (ELISA) in macrophages subjected to phagocytosis experiments showing that siRNA transfection does not increase anomalously the amount for IFN-γ. Results suggest that the observed phenotype is not a result of non-specific activation of macrophages with siRNA. ***B***: Cell dispersion pattern of control cells (EGFP), Bin2-EGFP (Bin2) and siRNA treated cells (siRNA) indicating that cells are similar in size and thus, the different uptake is not due to differences in cell size. FSC/SSC: forward/side scattered light, H: height of signal. ***C***: Phagocytosis assay (phagocytic index; expressed as the change in geometric mean fluorescence of positive cells compared with unstimulated cells) performed by incubated rat alveolar macrophages with ef670-labelled *S. pneumoniae* (Pn) or antibody-opsonised, immune complexed *S. pneumoniae* (Pn-IC) for 120 min at 37°C before analysis by flow cytometry. Uptake by Bin2-depleted cells (siRNA) or control siRNA and cells over-expressing Bin2-EGFP (Bin2) or EGFP was assessed. Data are the mean ± SD. Significance was calculated using the Student’s t test.
(TIF)

Movie S1 **Bin2 siRNA silenced mast cells (RBL·2H3) transiently overexpressing siRNA-insensitive rBin2 mutant.** A frame was acquired every second. This animation moves at 10 frames per second.
(MP4)

Movies S2 **Bin2 siRNA silenced mast cells (RBL·2H3) transiently overexpressing siRNA-insensitive rBin2 N-BAR mutant.** A frame was acquired every second. This animation moves at 10 frames per second.
(MP4)
