# Correction: Bin2 Is a Membrane Sculpting N-BAR Protein That Influences Leucocyte Podosomes, Motility and Phagocytosis

**DOI:** 10.1371/annotation/3bdc487b-5e25-4cd7-a354-b2952eec943d

**Published:** 2013-08-08

**Authors:** María José Sánchez-Barrena, Yvonne Vallis, Menna R. Clatworthy, Gary J. Doherty, Dmitry B. Veprintsev, Philip R. Evans, Harvey T. McMahon

Information concerning the the first author's funding was incorrectly omitted from the first sentence of the funding statement. The correct sentence is: MJS-B was funded by a FEBS postdoctoral fellowship during her stay in Cambridge (UK) and by a “Ramón y Cajal” contract (RYC-2008-03449) and the “Ministerio de Economía y Competitividad” (grant number BIO2011-28184-C02-02) in Spain.

