# Supplementary figures and images for: Bin2 Is a Membrane Sculpting N-BAR Protein That Influences Leucocyte Podosomes, Motility and Phagocytosis
Source: PLoS One. 2012 Dec 20;7(12):e52401. doi: 10.1371/journal.pone.0052401 (PMC3527510; doi:10.1371/journal.pone.0052401)

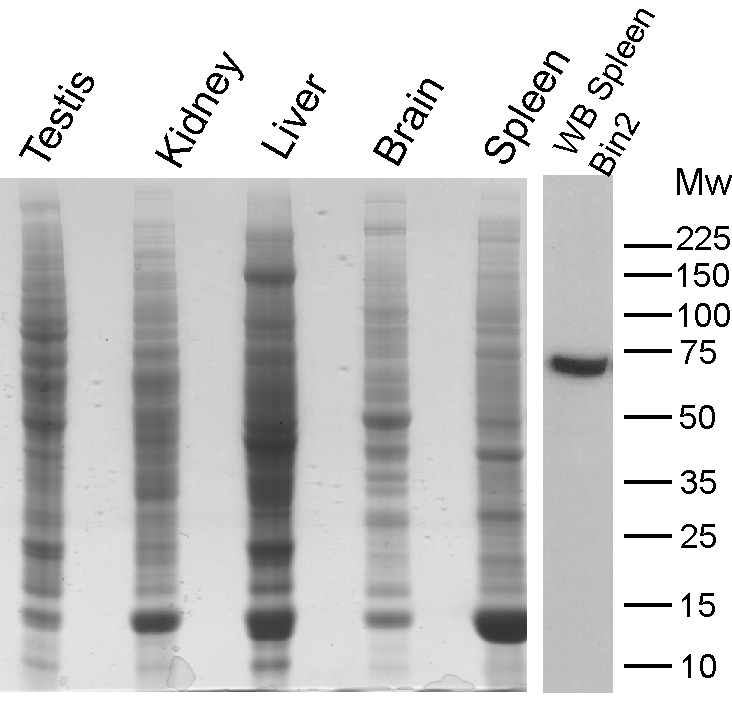

Supplement: Figure S1 — Bin2 is mainly expressed in leucocyte-enriched tissues. SDS-PAGE gel showing the amount of loaded protein from cell homogenates of different rat tissues. Right panel shows an antibody blot from spleen extract, showing the antibody detects a single protein species at approximately 70 kDa. The predicted mass of rBin2 is 60 kDa. Molecular weight markers (Broad Range, Promega) are indicated. (TIF) [file pone.0052401.s001.tif]

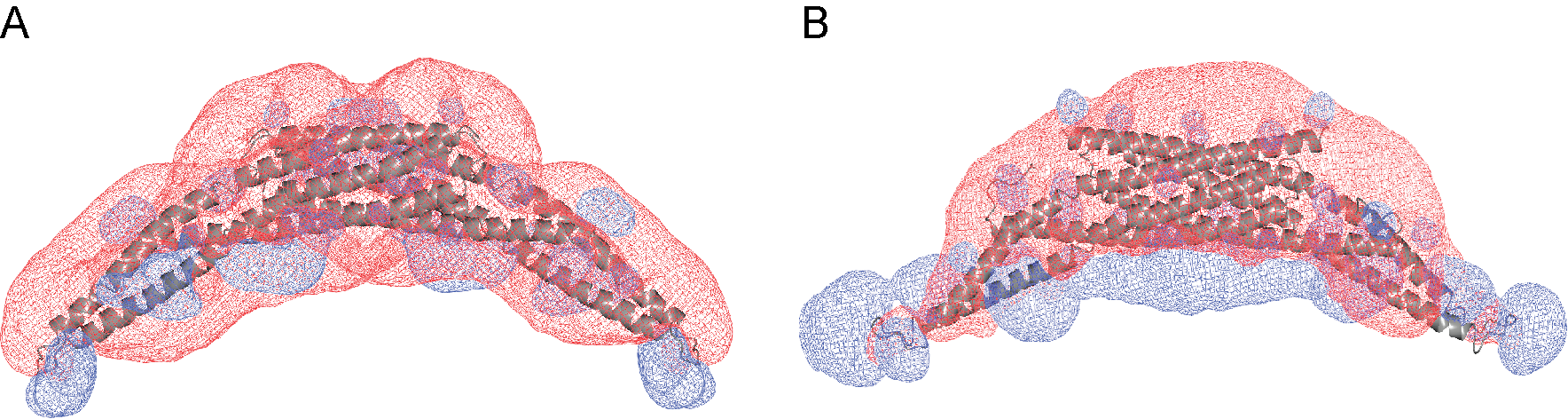

Supplement: Figure S3 — Electrostatic properties of hBin2, A , and dAmph, B , N-BARs. Equipotential surface representation contoured at 0.05V. Red and blue stand for negative and positive charges respectively. (TIF) [file pone.0052401.s003.tif]

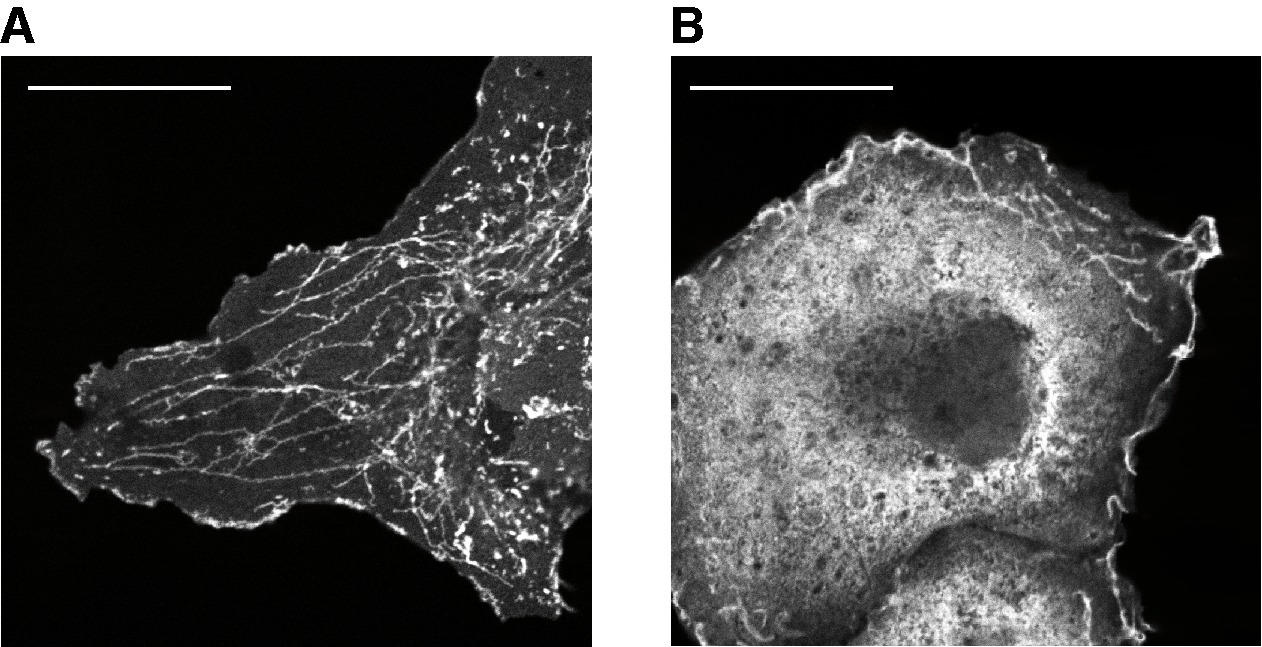

Supplement: Figure S4 — Bin2 tubulates membranes in vivo . Epifluorescent micrographs of fixed COS-7 cells transiently overexpression hBin2-EGFP, A, and Myc-hBin2, B. Scale bars, 20 µm. (TIF) [file pone.0052401.s004.tif]

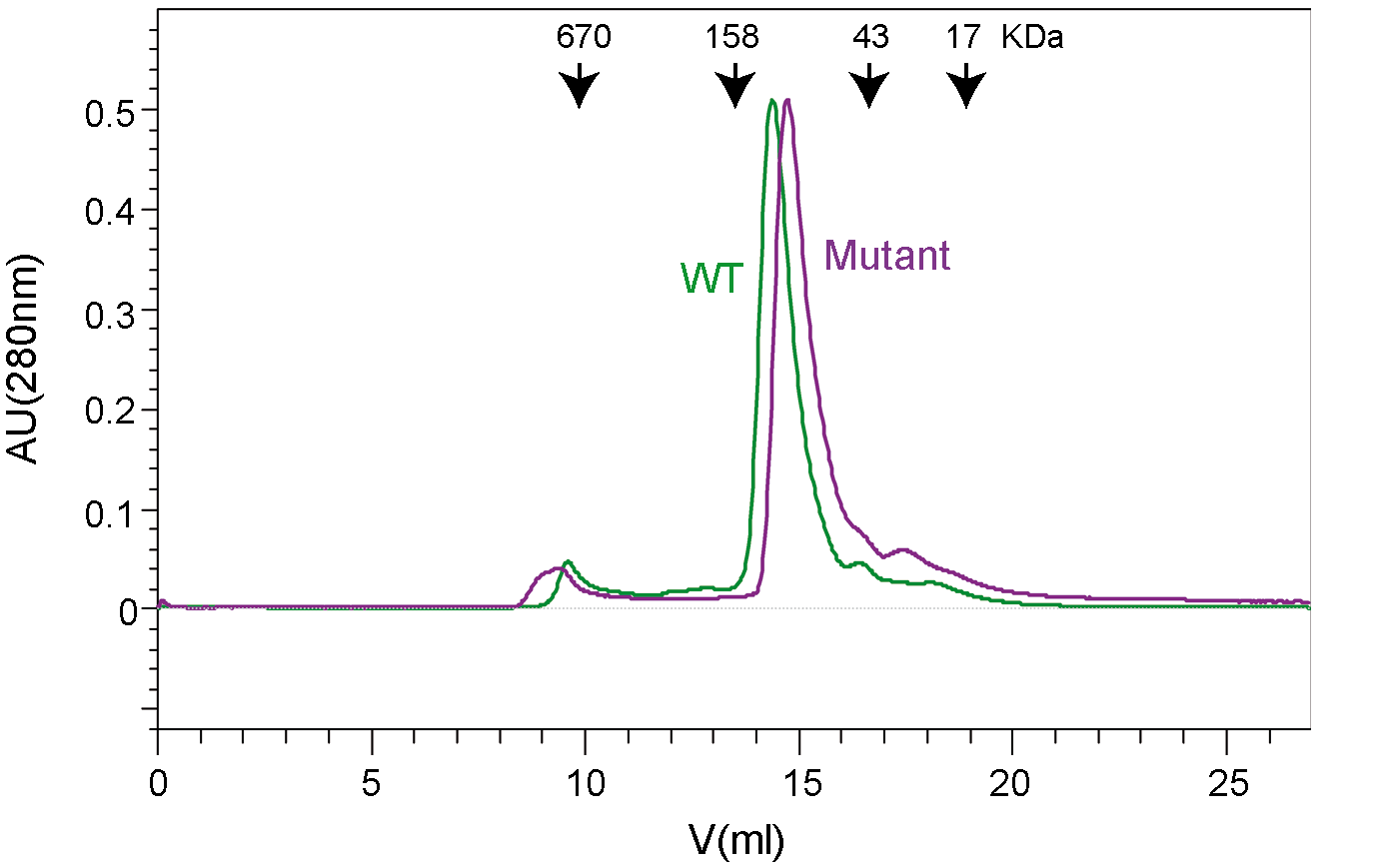

Supplement: Figure S5 — Gel filtration profiles of WT rBin2 N-BAR (green) vs rBin2 N-BAR V81R,S214E mutant (purple). Molecular weight standards (BioRad) are indicated with arrows. (TIF) [file pone.0052401.s005.tif]

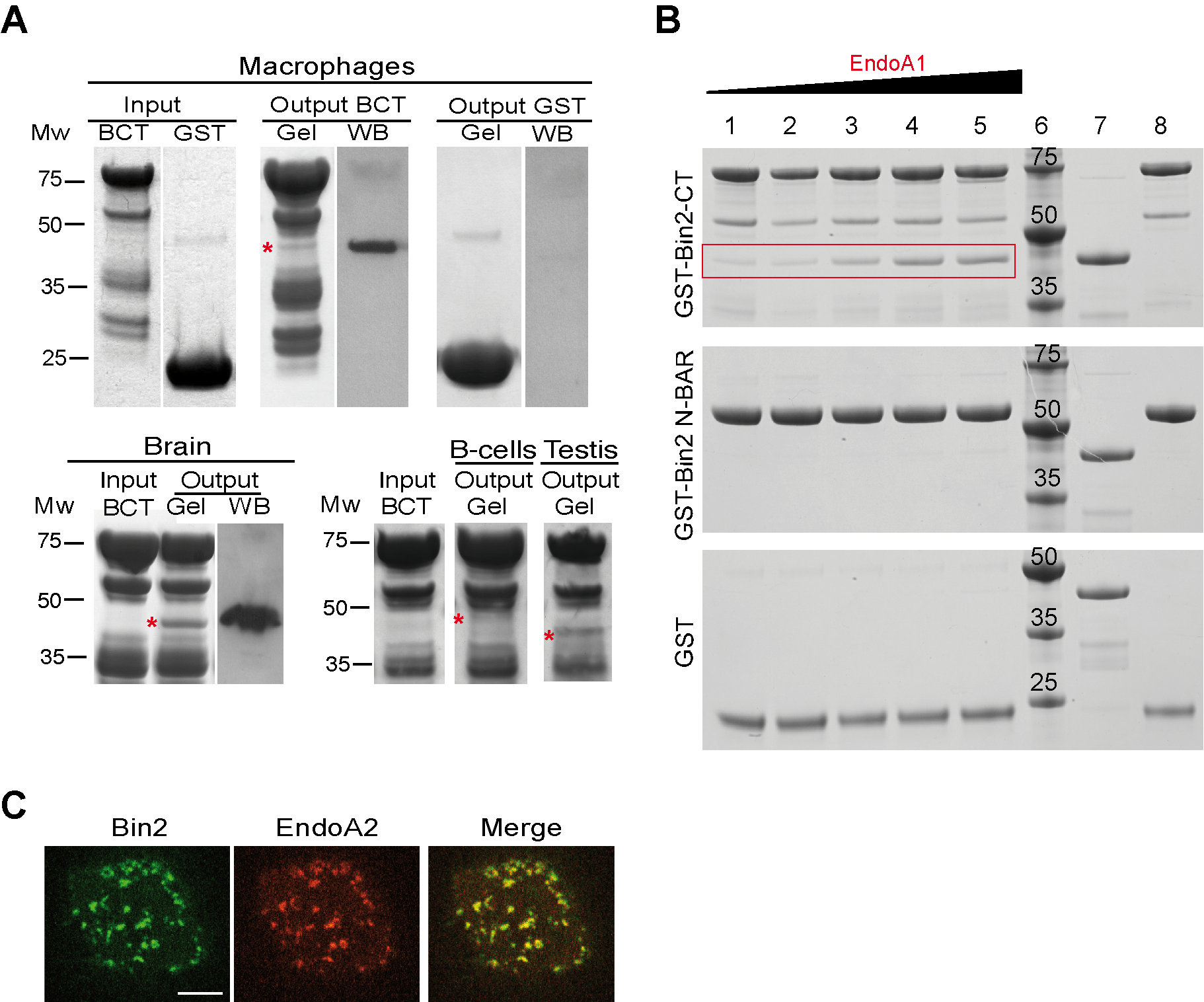

Supplement: Figure S6 — Bin2 interacts with Endophilin A2 at podosomes. A: Bin2 interacts with Endophilin A (EndoA) proteins. Pull-down assays using lymphocytes (human B cells and mouse macrophages), rat brain and rat testis cytosolic protein extracts. GST-hBin2-CT (238-end) (BCT) was immobilized on glutathione sepharose beads (Input). EndoA2 was pulled-down from B cell and macrophage cell extracts, EndoA1 from brain and Endophilin A3 from testis. The stars show the new bands that appeared after incubation with cellular protein extract (Output). Control experiments with GST (GST) were also performed. Bands were identified by LC-MS/MS and by western blot with the corresponding anti-Pan-EndoA, anti-EndoA1 and anti-EndoA2 Abs (Invitrogen). B: Bin2 interacts directly with EndoA proteins. In vitro pull-down assays with GST/GST-hBin2 (N-BAR and CT) and rEndoA1. In lanes 1–5 increasing amounts of EndoA1 were added to the GST-Bin2 pulldowns (red box). Lane 6, molecular weight markers (Broad range, Promega). Molecular weights (kDa) are indicated. Lane 7, EndoA1 alone. Lane 8, GST-Bin2-CT/NT or GST. C: EndoA2 colocalizes with Bin2 at the ring-like structure of podosomes. Basal surface images of rat mast cells (RBL·2H3) transiently expressing rBin2-EGFP and rEndoA2-mCherry. Scale bar, 5µm. (TIF) [file pone.0052401.s006.tif]

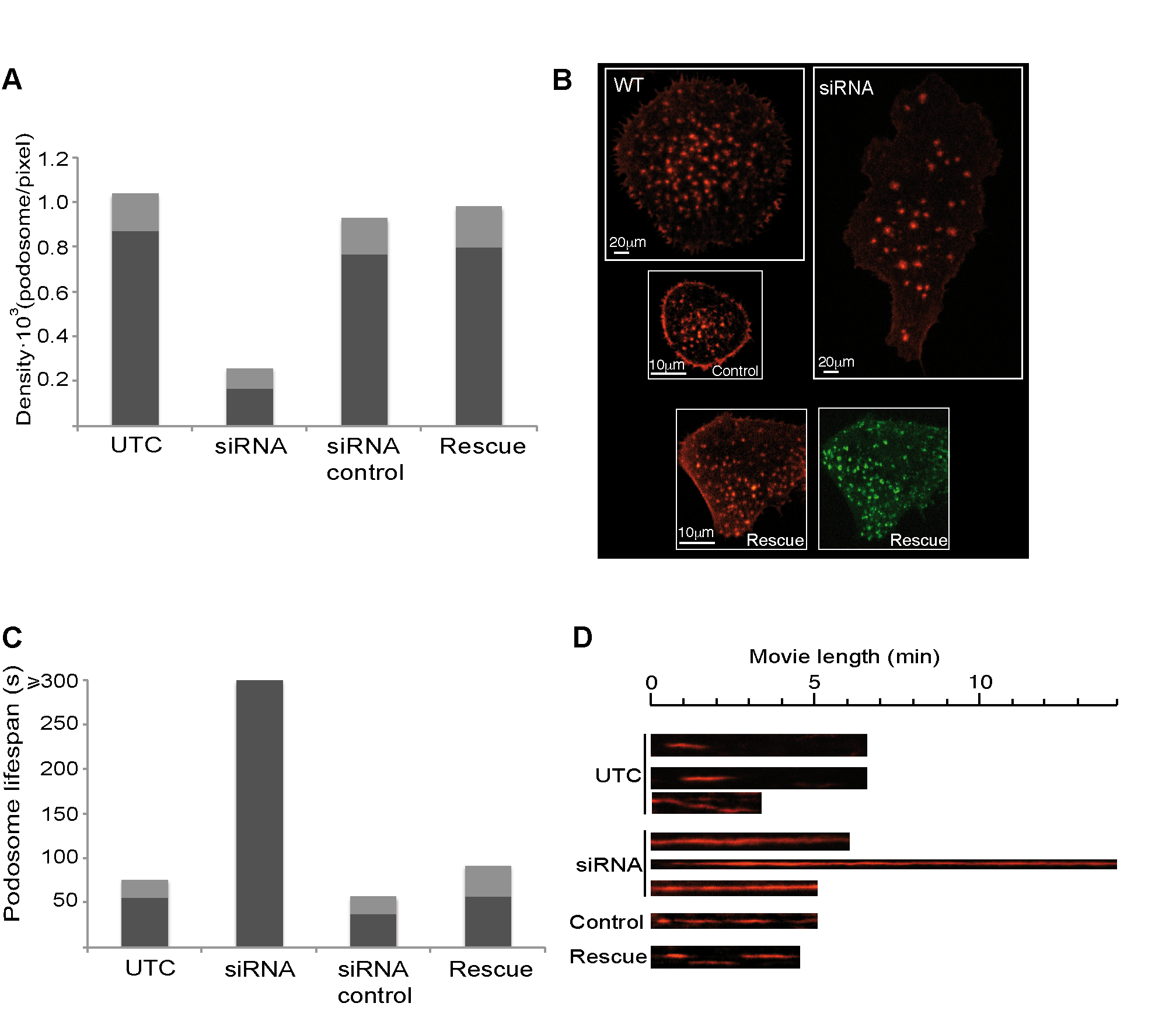

Supplement: Figure S7 — Bin2 influences podosome density and lifespan in mast cells (RBL·2H3). A: Bin2-depleted cells (siRNA) show a decreased density of podosomes with respect to WT (UTC) or siRNA-control cells (siRNA control). Overexpression of a siRNA-insensitive protein in Bin2 depleted cells rescues the phenotype. Light-grey bars show the standard deviation from the mean. B: Basal surface images of live cells. F-actin is labelled by LifeAct-mCherry (red) while Bin2-EGFP is in green. C: Podosomes from Bin2-silenced cells are not dynamic. D: Representative examples of kymographs showing the lifespan of podosomes. The length of the movies is indicated. (TIF) [file pone.0052401.s007.tif]

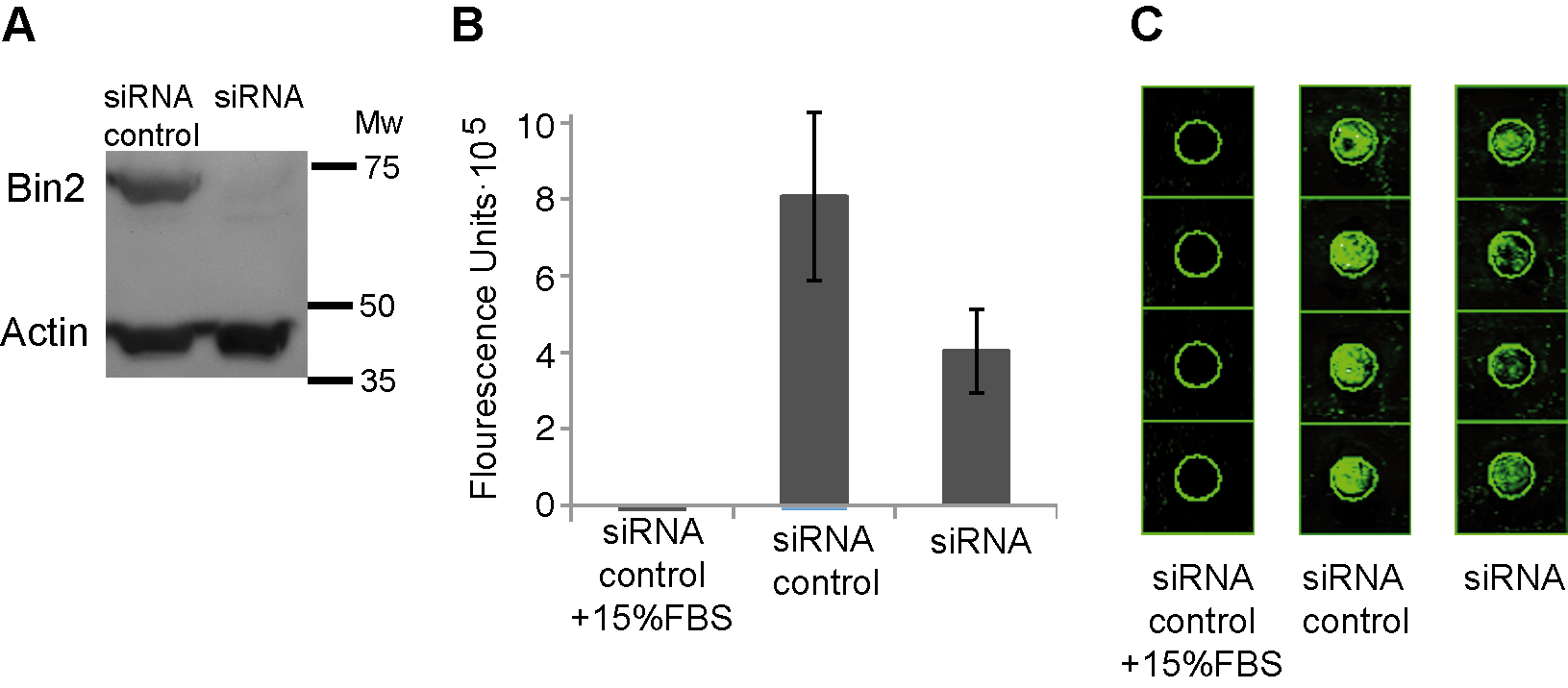

Supplement: Figure S8 — Bin2 regulates mast cell (RBL·2H3) migration. A: Western blot showing the effective depletion of rBin2 when cells are treated with siRNA. Actin was used as a loading control. Molecular weight markers (Broad Range, Promega) are indicated. B: Transwell migration of cells transfected with siRNA control vs siRNA under stimulating conditions (addition of 20 µg/ml fibronectin). siRNA control cells were also treated with 15% FBS to inhibit cell migration. Amount of migrating cells is expressed as fluorescence units. Data are the mean ± SD. C: Wells showing the amount of cells that were able to migrate during the experiment. Cells were stained with HCS CellMask (Invitrogen) for proper visualization. (TIF) [file pone.0052401.s008.tif]

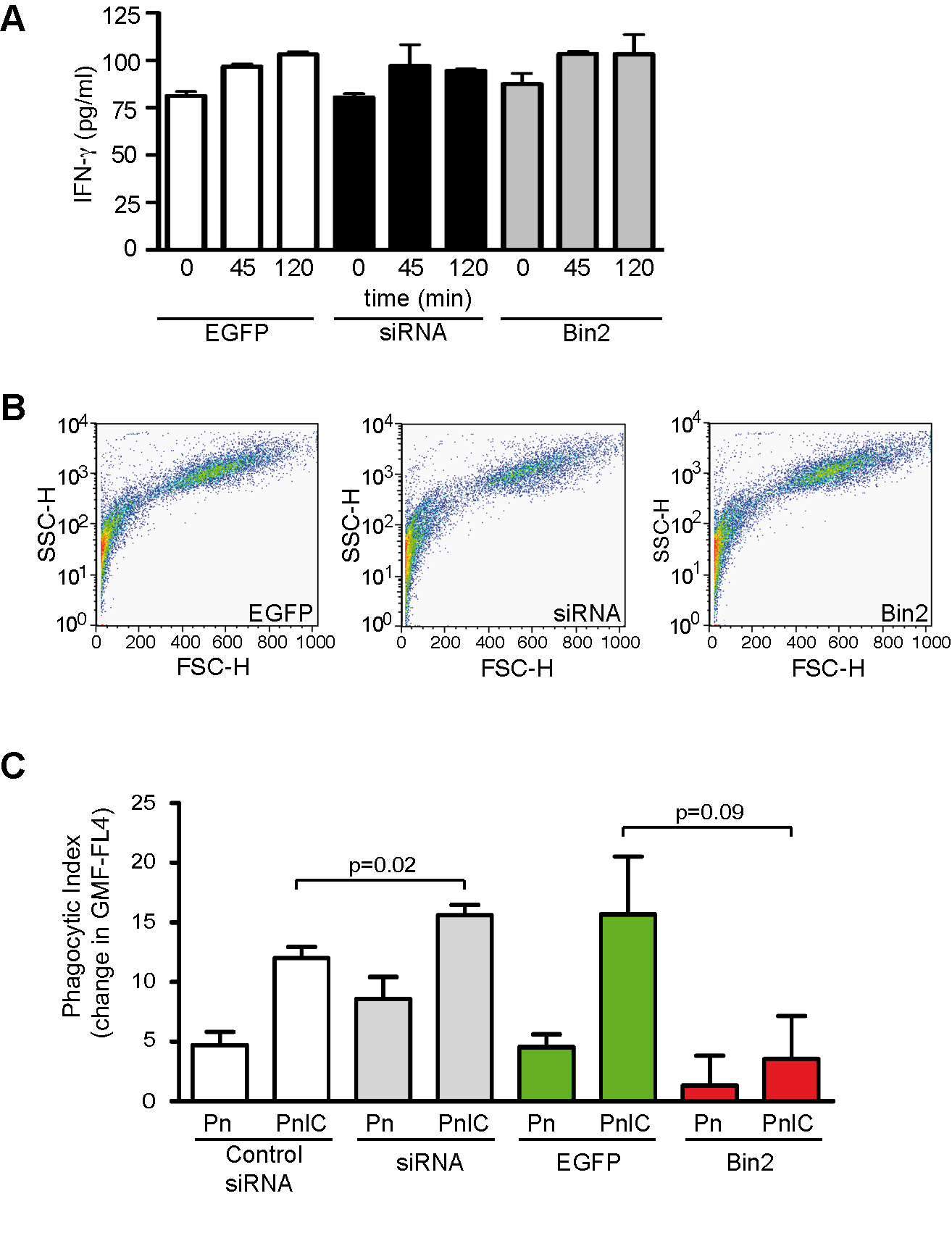

Supplement: Figure S9 — Phagocytosis assays. A: Levels of Interferon γ (IFN-γ) production (ELISA) in macrophages subjected to phagocytosis experiments showing that siRNA transfection does not increase anomalously the amount for IFN-γ. Results suggest that the observed phenotype is not a result of non-specific activation of macrophages with siRNA. B: Cell dispersion pattern of control cells (EGFP), Bin2-EGFP (Bin2) and siRNA treated cells (siRNA) indicating that cells are similar in size and thus, the different uptake is not due to differences in cell size. FSC/SSC: forward/side scattered light, H: height of signal. C: Phagocytosis assay (phagocytic index; expressed as the change in geometric mean fluorescence of positive cells compared with unstimulated cells) performed by incubated rat alveolar macrophages with ef670-labelled S. pneumoniae (Pn) or antibody-opsonised, immune complexed S. pneumoniae (Pn-IC) for 120 min at 37°C before analysis by flow cytometry. Uptake by Bin2-depleted cells (siRNA) or control siRNA and cells over-expressing Bin2-EGFP (Bin2) or EGFP was assessed. Data are the mean ± SD. Significance was calculated using the Student’s t test. (TIF) [file pone.0052401.s009.tif]
